# New Cyclic Diarylheptanoids from *Platycarya strobilacea*

**DOI:** 10.3390/molecules25246034

**Published:** 2020-12-20

**Authors:** Wen-bing Ding, Rui-yuan Zhao, Guan-hua Li, Bing-lei Liu, Hua-liang He, Lin Qiu, Jin Xue, You-zhi Li

**Affiliations:** 1 Hunan Provincial Engineering and Technology Research Center for Biopesticide and Formulation Processing, Hunan Agricultural University, Changsha 410128, China; dingwenb119@hunau.edu.cn; 2 National Research Center of Engineering and Technology for Utilization of Botanical Functional Ingredients, Hunan Agricultural University, Changsha 410128, China; lgh202@126.com (G.-h.L.); hhl_1234@126.com (H.-l.H.); qiulin@hunau.edu.cn (L.Q.); xuejin@hunau.edu.cn (J.X.); 3 Hunnan Cotton Science Institute, Changde 415100, China; zhaoruiyuan@vip.sina.com (R.-y.Z.); lbl5155@163.com (B.-l.L.)

**Keywords:** cyclic diarylheptanoid, *Platycarya strobilacea*, Juglandaceae, corticosterone-induced apoptosis

## Abstract

Five new cyclic diarylheptanoids (platycary A–E, compounds **1**–**5**) and three previously identified analogues (i.e., phttyearynol (compound **6**), myricatomentogenin (compound **7**), and juglanin D (compound **8**)) were isolated from the stem bark of *Platycarya strobilacea*. The structures of these compounds were determined using NMR, HRESIMS, and electronic circular dichroism (ECD) data. The cytotoxicity of compounds **1**–**5** and their ability to inhibit nitric oxide (NO) production, as well as protect against the corticosterone-induced apoptosis of Pheochromocytoma (PC12) cells, were evaluated in vitro using the appropriate bioassays. Compounds **1** and **2** significantly inhibited the corticosterone-induced apoptosis of PC12 cells at a concentration of 20 μΜ.

## 1. Introduction

More than 500 diarylheptanoids, an important class of plant secondary metabolites with a aryl-C7-aryl skeleton (linear or cyclic type), have been isolated from a number of plant families, including the Aceraceae, Actinidiaceae, Betulaceae, Burseraceae, Casuarinaceae, Zingiberaceae Leguminosae, Myricaceae, and Juglandaceae [1]. Cyclic diarylheptanoids are further divided into *meta*,*meta*-bridged biphenyls and *meta*,*para*-bridged diphenyl ether heptanoids, many of which have beneficial biological activities such as anti-tumor, anti-inflammatory, estrogenic, anti-amyloidogenic, and anti-emetic activity [1,2]. Hence, the discovery of new natural diarylheptanoids and their phytochemical characterization is of considerable potential benefit to medical science. *Platycarya strobilacea* Sieb. et Zucc. (Juglandaceae) is a deciduous shrubby tree distributed in East Asia [3]. Previous phytochemical studies have found that this species contains large amounts of ellagitannins [4], flavonoids [5], and sesquiterpenoids [6,7], but apparently only one cyclic diarylheptanoid (i.e., platycarynol) [8]. Since a number of cyclic diarylheptanoids have been isolated from many species of Juglandaceae, in this paper we described the results of an extensive investigation of the stem bark of *P. strobilacea* in which eight cyclic diarylheptanoids, including five new ones (compounds **1**–**5**), were isolated and identified. Furthermore, the anti-tumor cytotoxic activity of the five new isolates, and their ability to inhibit nitric oxide (NO) production and the corticosterone-induced apoptosis of PC12 cells, was evaluated in vitro.

## 2. Results and Discussion

**Figure 1:**
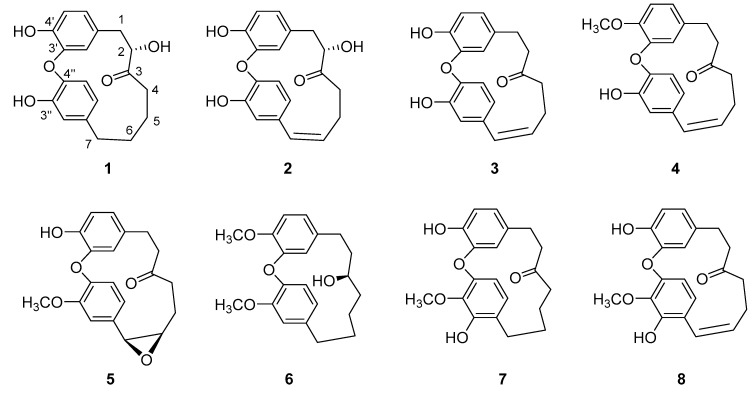
Structures of compounds **1**–**8**.

A 95% EtOH extract of the stem bark of *P. strobilacea* was subjected to column chromatography (CC) over silica gel, Sephadex LH-20, and pre-TLC to afford compounds **1**–**8**. The structures of the known compounds phttyearynol (**6**), myricatomentogenin (**7**), and juglanin D (**8**) were determined by spectral data analysis and comparison with published data (Figure 1) [8,9,10].

**Figure 2:**
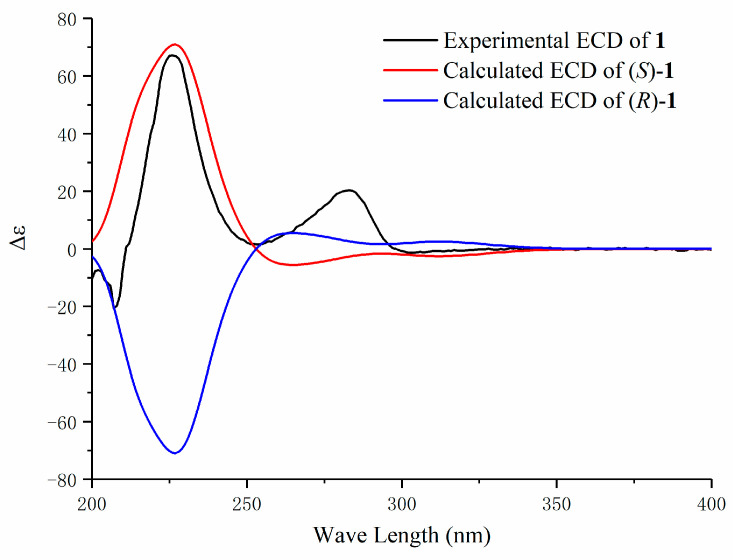
Experimental and calculated electronic circular dichroism (ECD) spectra of compound **1.**

Compound **1** was isolated as a white solid. Its molecular formula was assigned as C_19_H_20_O_5_ by the HRESIMS (*m*/*z* 327.1234 [M − H]^−^, calcd for C_19_H_19_O_5_, 327.1232). The ^13^C-NMR spectrum exhibited 19 carbon signals including characteristic peaks of a carbonyl group and 12 aromatic carbons (see the Supplementary Materials); the other carbons were identified using the HSQC spectrum as an oxymethine and five methylene (Table 1 and Table 2). Detailed analysis using 2D NMR spectra revealed a heptanoid chain (–1-CH_2_–2-CH_2_OH–3-(C=O)–4-CH_2_–5-CH_2_–6-CH_2_–7-CH_2_–) moiety established by the ^1^H-^1^H COSY cross peaks of H-1/H-2, H-4/H-5, H-5/H-6, and H-6/H-7, as well as the Heteronuclear Multiple Bond Correlation (HMBC) cross peaks of H-1/C-3, H-2/C-3, H-2/C-4, and H-5/C-3. In the ^1^H-NMR spectrum, six aromatic proton signals at *δ*_H_ 6.57 (dd, *J* = 8.1, 2.1 Hz, H-6′), 5.72 (d, *J* = 2.1 Hz, H-2′), 6.72 (d, *J* = 8.1 Hz, H-5′), 6.82 (d, *J* = 2.0 Hz, H-2″), 6.77 (dd, *J* = 8.2, 2.0 Hz, H-6″), and 6.83 (d, *J* = 8.2 Hz, H-5″) revealed the presence of two 1,3,4-trisubstituted aromatic rings that were ABX coupling patterns. The chemical shift of H-2′ at 5.72 ppm appeared highly upfield from other aromatic proton signals, and such a shielding effect is characteristic of biaryl-type cyclic diarylheptanoids that have an ether linkage between C-3′ and C-4″ [2,8,11]. The above information suggests that compound **1** is 2-hydroxy-3′,4″-epoxy-1-(4′-hydroxyphenyl)-7-(3″-hydroxyphenyl)-3-heptanone. The cross-peaks between H-1 and C-1′, C-2′, and C-6′, and those between H-7 and C-1″, C-2″, and C-6″ in the HMBC spectrum confirmed the linkages of the heptanoid chain with two benzene rings. For the absolute configuration of the hydroxyl group at C-2, the theoretical calculations of electronic circular dichroism (ECD) for *R* and *S*-conformers were conducted in MeOH using TD-DFT at the B3LYP/6-31+g (d, p) level (performed using the Gaussian 09 program) [12]. The experimental ECD spectrum of compound **1** was compared with what was calculated using this method (Figure 2) and was determined to be a good match to the 2*S*-configuration. Moreover, the positive optical rotation ((α)${}_{D}^{25}$ + 143.33 (*c* 0.06, MeOH)) of compound **1** was opposite to that of 2(*R*)-hydroxy-3′,4″-epoxy-1-(4′-hydroxyphenyl)-7-(3″-methoxylphenyl)-3-heptanone ((α)${}_{D}^{25}$ − 81.13(*c* 0.03, MeOH)), a very similar structure previously isolated from the roots of *Juglans mandshurica* [11]. Therefore, the structure of compound **1** was determined (Figure 1) and named platycary A.

**Figure 3:**
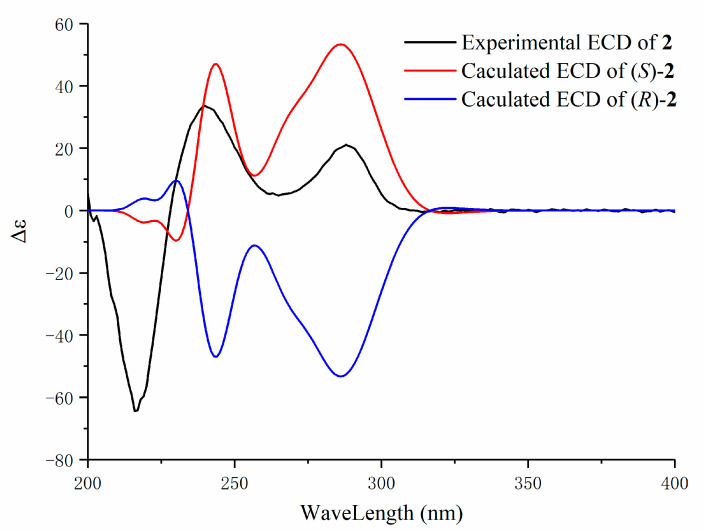
Experimental and calculated ECD spectra of compound **2**.

Compound **2** was isolated as a white solid. Its molecular formula was assigned as C_19_H_18_O_5_ by the HRESIMS (*m/z* 325.1073 [M − H]^−^, calcd for C_19_H_17_O_5_, 325.1076). Its ^13^C-NMR spectrum exhibited a total of 19 carbon signals, including the characteristic peaks of a carbonyl group, a hydroxyl carbon, and 12 aromatic carbons as seen in compound **1**, and two groups of ABX patterns aromatic proton signals including the highly upfield H-2′ (*δ*_H_ 5.57, br s), which was also observed in the ^1^H-NMR spectrum. These spectroscopic characteristics suggest that compound **2** is a biaryl-type cyclic diaryheptanoid. However, rather than the two methylene signals found in compound **1**, two additional *cis*-olefinic proton signals at *δ*_H_ 6.75 (d, *J* = 11.2 Hz, H-7) and 5.99 (dt, *J* = 11.2, 7.2 Hz, H-6), as well as the corresponding carbon signals at 132.2 and 133.2 ppm, were apparent in compound **2.** Further detailed analysis of compound **2** using 2D NMR spectra indicated a different heptanoid chain (-1-CH_2_–2-CH_2_OH–3-(C=O)–4-CH_2_–5-CH_2_–6-CH=7-CH–). The double bond at C-6 and C-7 was established by the ^1^H-^1^H COSY correlations of H-4/H-5, H-5/H-6, H-6/H-7, and the HMBC correlations of H-6/C-1″ and H-7/C-1″, C-2″, C-6″. Moreover, the absolute configuration of C-2 was assigned to be *S* by comparison with ECD cotton effects (Figure 3). Finally, the structure of compound **2** was proposed and named platycary B.

Compound **3** has the molecular formula C_19_H_18_O_4_, determined from HRESIMS and NMR data. The ^1^H-NMR spectrum of compound **3** was very similar to that of compound **2**, with the same upfield aromatic signal of H-2′ (*δ*_H_ 5.38, d) and the ABX coupling pattern due to two sets of 1,3,4-trisubstituted aromatic groups. However, in compound **3**, the hydroxyl carbon signal (*δ*_C_ 76.6, C-2 in compound **2**) of the heptanoid was replaced by a methylene signal (*δ*_C_ 40.8, C-2). Cross-peaks in the ^1^H-^1^H COSY and HMBC spectra indicate connectivity from H-1 to H-7, creating a straight chain of the hept-6-en-3-one moiety. Moreover, the linkages of this chain to the two benzene rings were established by the HMBC cross-peaks of H-1/C-1′, C-2′, and C-6′, as well as those H-7/C-1″, C-2″, and C-6″. Thus, the structure of compound **3** was determined (as shown in Figure 1) and named platycary C.

Compound **4** has the molecular formula C_20_H_20_O_4,_ determined from HRESIMS and NMR data. Its ^1^H and ^13^C-NMR data (Table 1 and Table 2) are quite similar to that of compound **3**, except for an additional methoxyl moiety (*δ*_C_ 56.1), and slight differences in the chemical shifts of C-3′, C-4′, and C-5′ that hint to the linkage site of the methoxyl group. The HMBC correlations between -OC*H*_3_ and C-4′ further verified that the methoxyl group was located at C-4′. Therefore, the structure of compound **4** was proposed and named platycary D.

Compound **5** was isolated as white needle-shaped crystals. Its molecular formula was deduced to be C_20_H_20_O_5_ using HRESIMS (*m/z* 339.1232 [M − H]^−^, calcd for 339.1232). The ^1^H and ^13^C-NMR spectra of compound **5** (Table 1 and Table 2) exhibited characteristic signals of a carbonyl group, a methoxyl group, and two 1,3,4-trisubstituted phenyl groups (as were also apparent for compound **4**), while the difference between compound **5** and compound **4** was the heptanoid chain. Detailed analysis of the NMR data of compound **5** revealed two new signals at *δ*_C_ 55.9 and 56.2 ppm, which are typical indicators of epoxide functionality [13,14], whereas two olefinic carbons (*δ*_C_ 131.6 and 131.9) were present in compound **4**. The expected 6,7-epoxy-heptan-3-one moiety was established using ^1^H-^1^H COSY and HMBC correlations. In addition, the linkage site of the methoxyl group was assigned to C-3″ by the HMBC correlation of OC*H*_3_/C-3″. The relative stereochemistry of the epoxide was determined as *cis* based on the coupling constant between H-6 and H-7 (*J* = 4.3 Hz) (13,14), which was confirmed by a strong cross peak between H-6 and H-7 in the NOESY spectrum. Therefore, the structure of compound **5** was proposed and named platycary E.

**Figure 4:**
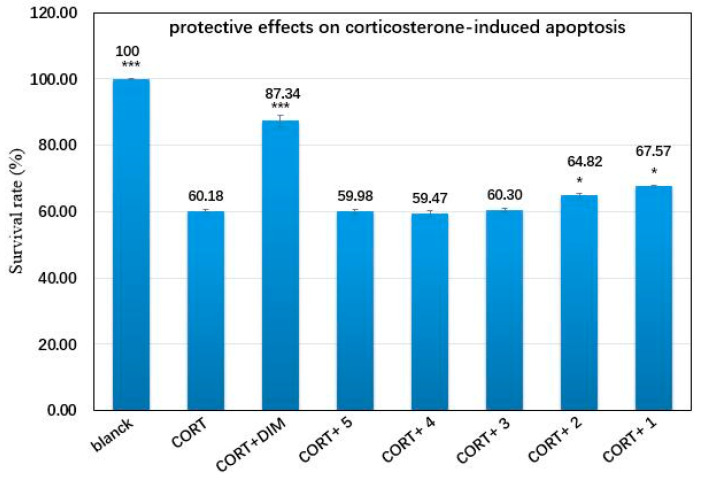
Protective effects of compounds **1**–**5** against corticosterone-induced apoptosis in PC12 cells. CORT = corticosterone, DIM = desipramine. Paired t-tests were used to evaluate the statistical significance of differences between different treatment groups and the model group (CORT). * Represents a *p*-value of < 0.05, *** represents a *p*-value of < 0.001.

Previously, Jahng and Park (2018) summarized series of cyclic diaryl ether heptanoids and their biological properties. Some of these compounds showed cytotoxic activities against human cancer cell lines, nitric oxide (NO) production inhibitory activity, neuroprotective activity, radical scavenging activity, and osteogenic activity [2]. In view of the valuable pharmacological activities of cyclic diarylheptanoids, we tested the cytotoxic activity of compounds **1**–**5** against human cancer cell lines (including HL-60, A549, SMMC-7721, MCF-7, and SW480), and their ability to inhibit NO production in Lipopolysaccharide (LPS) activated murine macrophage RAW 264.7 cells and protective effect of corticosterone-induced apoptosis in Pheochromocytoma (PC12) cells. The results indicated that none of the five compounds showed cytotoxic activities (at 40 μM) or inhibited NO production (at 50 μM). However, compounds **1** and **2** significantly inhibited the corticosterone-induced apoptosis of PC12 cells at the concentration of 20 μΜ (Figure 4). The PC12 cell line is the most commonly used neuronal cell line for neuroscience research in vitro studies. A previous study indicated that corticosterone (CORT) at high concentrations induces cell injury and cell death in PC12 cells, and desipramine (DIM) can antagonize the neurotoxicity caused by CORT [15]. Our report indicated that compounds **1** and **2** are two potential cytoprotective agents for neurological diseases associated with corticosterone-induced neurotoxicity.

## 3. Materials and Methods 

### 3.1. General

Melting points were obtained on a SGW X-4 micro melting point apparatus (INESA Co., Shanghai, China). Optical rotations were measured on a SGW-533 automatic polarimeter (INESA Co., Shanghai, China). The 1D and 2D NMR spectra were measured with a Bruker DRX-600 instrument (Bruker BioS-pin GmbH Company, Rheinstetten, Germany) with TMS as the internal standard. ESIMS data were recorded on an API QSTAR mass spectrometer (Applied Biosystem/MSD Sciex, Concord, Ontario, Canada); HRESIMS were recorded on a Waters Xevo G2-XS QTof mass spectrometer (USA). ECD spectra were recorded on a JASCO J-810 circular dichroism (CD) spectropolarimeter (JASCO Co., Tokyo, Japan). Column chromatography was performed on silica gel 60 (100–200 mesh, Qingdao Marine Chemical Ltd., Qingdao, China), sephadex LH-20 (GE Healthcare, Uppsala, Sweden), and Develosil ODS (50 μm, Nomura Chemical Co. Ltd., Osaka, Japan). The preparation of TLC was performed on HSGF254 plates (Jiangyou silicone Development Co., Ltd., Yantai, China).

### 3.2. Plant Material

The stem bark of *P. strobilacea* was collected from Cili county, Hunan Province, P.R. China (29°42′ N and 111°13′ E) in August 2018, and identified by Professor Zhang Dai-gui (Key Laboratory of Plant Resources Conservation and Utilization, Jishou University). A voucher specimen (zdg20180809) was deposited at the College of Plant Protection, Hunan Agricultural University.

### 3.3. Extraction and Isolation 

Dried barks of *P. strobilacea* (12.5 kg) were powdered and extracted three times with EtOH (95% *v*/*v*) at room temperature, then concentrated under reduced pressure to obtain a crude residue (1.8 kg). The residue was suspended in H_2_O (4 L) and then extracted with petroleum ether (PE) and EtOAc successively, to yield a PE-soluble fraction (35.0 g) and an EtOAc-soluble fraction (95.0 g). The EtOAc-soluble fraction was further subjected to silica gel CC (100–200 mesh) with elution of CHCl_3_-MeOH (100:0 → 60:40, *v*/*v*) to give seven fractions (Fr. A–G). Fr.B (5.7 g) was decolorized on MCI gel (eluted with 90 % MeOH) and then chromatographed on an ODS-C18 column that eluted with acetone-H_2_O (3:7→10:0, *v*/*v*) to afford 10 sub-fractions (B1−B10). After TLC detection, sub-fractions B5, B6, B7, B9, and B10 with clear spots were further purified by Sephadex LH-20 column chromatography (eluted with MeOH). Compounds **5** (15.2 mg), **6** (23.7 mg) and **8** (10.8 mg) were obtained from sub-fractions B5, B6, and B10 by preparation TLC using CHCl_3_-MeOH (95:5) as a developing solvent, respectively. Compounds **7** (20.5 mg) and **3** (117.8 mg) were obtained from sub-fractions B7 via methanol recrystallization, while compound **4** (50.8 mg) was yielded from sub-fraction B9 via recrystallization (CHCl_3_-MeOH, 1:1 *v*/*v*). Similarly, Fr.C (6.4 g) was successively subjected on MCI gel and ODS-C18 column to afford 10 sub-fractions (C1−C10). Compounds **1** (27.9 mg) and **2** (8.0 mg) were obtained from sub-fractions C6 using methanol recrystallization and the prepared TLC using CHCl_3_-MeOH (95:5) as a developing solvent.

### 3.4. Characterization of Compounds ***1**–**5***

Compound **1:** white solid, m.p. 183 °C, (*α*)${}_{D}^{25}$ +143.33 (*c* 0.06, MeOH); ECD data (MeOH): Δε (nm) = −20.71 (207), +67.18 (226), +20.25 (283). ^1^H-NMR (600 MHz, CD_3_OD) and ^13^C-NMR (150 MHz, CD_3_OD) spectroscopic data, see Table 1 and Table 2; positive ion ESIMS *m/z*: 329 [M + H]^+^, negative ESIMS *m/z*: 327 [M − H]^−^, 363 [M + Cl]^−^ HRESIMS *m*/*z*: 327.1234 [M − H]^−^ (calcd for C_19_H_19_O_5_, 327.1232).

Compound **2:** white solid, m.p. 194 °C, (α)${}_{D}^{25}$ +103.33 (*c* 0.12, MeOH); ECD data (MeOH): Δε (nm) = −80.68 (218), +67.63 (240), +41 (288). ^1^H-NMR (600 MHz, CD_3_OD) and ^13^C-NMR (150 MHz, CD_3_OD) spectroscopic data, see Table 1 and Table 2; positive ion ESIMS *m/z*: 327 [M + H]^+^, negative ESIMS *m/z*: 325 [M − H]^−^, 361 [M + Cl]^−^ HRESIMS *m*/*z*: 325.1073 [M − H]^−^ (calcd for C_19_H_17_O_5_, 325.1076).

Compound **3:** colorless crystal, m.p. 135 °C; ^1^H-NMR (600 MHz, CD_3_OD) and ^13^C-NMR (150 MHz, CD_3_OD) spectroscopic data, see Table 1 and Table 2; positive ion ESIMS *m/z*: 311 [M + H]^+^, negative ESIMS *m/z*: 309 [M − H] ^−^; HRESIMS *m*/*z*: 309.1129 [M − H]^−^ (calcd for C_19_H_17_O_4_, 309.1127).

Compound **4:** colorless crystal, m.p. 119 °C; ^1^H-NMR (600 MHz, CDCl_3_) and ^13^C-NMR (150 MHz, CDCl_3_) spectroscopic data (Table 1 and Table 2); 325 [M + H]^+^, 347 (M + Na]^+^; negative ESIMS *m/z*: 323 [M − H]^−^, 359 [M + Cl]^−^; HRESIMS *m*/*z*: 323.1280 [M − H]^−^ (calcd for C_20_H_19_O_4_, 323.1283).

Compound **5:** white needle-shaped crystal, m.p. 222 °C; ^1^H-NMR (600 MHz, CDCl_3_) and ^13^C-NMR (150 MHz, CDCl_3_) spectroscopic data (Table 1 and Table 2); 341 [M + H]^+^; negative ESIMS *m/z*: 339 [M − H]^−^, 375 [M + Cl]^−^; HRESIMS *m*/*z*: 339.1232 [M − H]^−^ (calcd for C_20_H_19_O_5_, 339.1232).

### 3.5. Determination of Biological Activity

The in vitro cytotoxicity of compounds **1**–**5** against human cancer cell lines (HL-60, A549, SMMC-7721, MCF-7, and SW480) was assayed using the 3-(4,5-dimethylthiazol-2-yl)-5(3-carboxymethoxyphenyl)-2-(4-sulfopheny)-2H-tetrazolium (MTS) method, wherein Taxol was used as the positive control [16,17]. 

The inhibitory effects of compounds **1**–**5** on NO production were evaluated in LPS-activated murine macrophage RAW 264.7 cells, using a method modified from what had been previously reported [18]. RAW264.7 was purchased from the Chinese Academy of Sciences’ Shanghai cell bank, and Dulbecco’s modified Eagle’s medium (DMEM) medium and fetal bovine serum were purchased from the Bi Company. Griess reagent, Lipopolysaccharide (LPS), and the comparator L-N^G^-Monomethyl Arginine citrate (L-NMMA) were purchased from Sigma-Aldrich (Steinheim, Germany).

The protective effects of compounds **1**–**5** on corticosterone-induced apoptosis were evaluated using a previously reported method [19]. Briefly, differentiated PC12 cells were maintained in DMEM medium supplemented with 10% fetal bovine serum (FBS), penicillin (100 U/mL), and streptomycin (100 μg/mL), and were incubated at 5% CO_2_ at 37 °C. Poorly differentiated PC12 cells were divided into the following groups: untreated, CORT (150 μmol/L), CORT (150 μmol/L) plus DIM (10 μmol/L) as the positive control, and CORT (150 μmol/L) plus one of the test compounds (20 μmol/L). Poorly differentiated PC12 cells were seeded into 96-well culture plates at a density of 1 × 104 cells/well. After a 24 h culture, the wells were added as compounds, as previously described. Next, 48 h later, MTS solution was added to each well. An absorbance was measured at 492 nm using a Thermo Multiskan FC. All data was expressed as mean ± SD and paired t-tests implemented in SPSS 13.0 were used to evaluate the statistical significance of the differences between separate treatment groups and the control.

The above biological activities tests were conducted at the Natural Drug Activity Screening Center, Kunming Institute of Botany, Chinese Academy of Sciences.

## 4. Conclusions

Phytochemical investigation of the stem bark of *P. strobilacea* afforded five new cyclic diarylheptanoids (i.e., platycary A−E (compounds **1**–**5**)) and three known ones: phttyearynol (compound **6**), myricatomentogenin (compound **7**), and juglanin D (compound **8**). Biological activities of compounds **1**–**5** were evaluated using cytotoxicity, NO, and corticosterone-induced apoptosis assays. Compounds **1** and **2** exhibited significant protective effects against the corticosterone-induced apoptosis of PC12 cells at the concentration of 20 μΜ, indicating that compounds **1** and **2** are two potential cytoprotective agents for neurological diseases. The identification of these new compounds significantly increases both the number of diarylheptanoids produced by *P. strobilacea* and the number of diarylheptanoids with potential medical value.

## Figures and Tables

**Table 1:** The ^1^H-NMR (600 MHz) data of compounds **1**–**5**, *δ*_H_ in ppm (coupling type, *J* in Hz).

No.	1 ^a^	2 ^a^	3 ^a^	4 ^b^	5 ^b^
1	3.07 (dd, 15.1, 2.0)	3.18 (d, 15.8)	2.87 (m)	3.00 (m)	3.25 (m)
	2.82 (dd, 15.1, 7.6)	2.91 (dd, 15.8, 7.6)	2.80 (m)	2.85 (dd, 16.4, 8.3)	2.51 (dd, 16.1, 5.4)
2	3.98 (dd, 7.6, 2.0)	4.06 (dd, 7.7, 1.9)	2.35–2.39 (m)	2.40 (dd, 17.4, 9.6)	2.27 (dd, 17.7, 5.4)
				2.33 (dd, 16.8, 7.7)	2.46 (m)
4	2.12 (m)	2.36 (m)	2.18–2.24 (m)	2.22 (m)	2.40 (m)
	1.64 (m)	2.27 (m)		2.09 (m)	1.52 (dd, 19.0, 11.5)
5	1.46–1.56 (m)	2.55 (m)	2.38 (m)	2.52 (m)	2.06 (m)
		2.14 (m)	2.31 (m)	2.28 (m)	1.71 (m)
6	1.75 (m)	5.99 (dt, 11.2, 7.2)	5.97 (dt, 11.5, 7.3)	5.97 (dt, 11.2, 7.2)	3.26 (m, overlapped)
	1.60 (m)				
7	2.75 (m)	6.75 (d, 11.2)	6.71 (d, 11.5)	6.71 (d, 11.2)	4.18 (d, 4.3)
	2.55 (m)				
2′	5.72 (d, 2.1)	5.57 (br s)	5.38 (d, 2.0)	5.45 (d, 2.0)	5.16 (d, 2.2)
5′	6.72 (d, 8.1)	6.76 (overlapped)	6.73 (d, 8.1)	6.82 (d, 8.1)	6.84 (d, 8.3)
6′	6.57 (dd, 8.1, 2.1)	6.59 (dd, 8.2, 2.1)	6.57 (dd, 8.1, 2.0)	6.73 (dd, 8.1, 2.0)	6.63 (dd, 8.3, 2.2)
2″	6.82 (d, 2.0)	6.87 (d, 1.9)	6.83 (d, 1.9)	6.90 (d, 2.0)	6.97 (d, 1.7)
5″	6.83 (d, 8.2)	6.96 (d, 8.2)	6.92 (d, 8.1)	6.99 (d, 8.2)	7.06 (overlapped)
6″	6.77 (dd, 8.2, 2.0)	6.73 (overlapped)	6.74 (dd, 8.1, 1.9)	6.83 (dd, 8.2, 2.0)	7.06 (overlapped)
4′-OCH_3_				3.91 (s)	
3″-OCH_3_					3.72 (s, 3H)

^a^ Data were measured in CD_3_OD; ^b^ Data were measured in CDCl_3_.

**Table 2:** The ^13^C-NMR (150 MHz) data of compounds **1**–**5**, *δ*_C_ in ppm**.**

NO.	1 ^a^	2 ^a^	3 ^a^	4 ^b^	5 ^b^
1	38.5	37.9	27.7	26.7	26.7
2	77.3	76.6	40.8	39.9	40.0
3	215.5	214.3	211.4	208.5	207.3
4	42.7	39.0	42.9	42.1	37.9
5	20.8	20.4	20.0	18.8	20.9
6	28.6	132.2	132.4	131.6	56.2
7	36.8	133.2	133.1	131.9	55.9
1′	141.2	138.4	138.5	137.3	134.4
2′	119.6	118.4	118.4	116.5	110.8
3′	151.5	151.7	151.7	149.2	152.5
4′	144.1	144.1	143.3	141.3	144.0
5′	125.3	125.5	125.5	123.8	124.0
6′	122.8	121.7	121.6	121.3	119.6
1″	129.4	129.5	133.8	133.8	132.4
2″	116.6	115.7	113.5	112.9	111.6
3″	149.4	149.3	149.4	148.7	147.5
4″	145.6	145.2	144.6	146.4	143.0
5″	117.0	117.2	117.2	112.1	115.3
6″	124.9	124.6	122.9	122.5	122.4
4′-OCH_3_				56.1	
3″-OCH_3_					56.2

^a^ Data were measured in CD_3_OD; ^b^ Data were measured in CDCl_3_.

## Supplementary Materials

The following are available online, Figures S1−S33 associated with the HRESIMS, ^1^H-NMR, ^13^C-NMR, and 2D NMR spectra of new compounds.
