# Supplementary material for: New Cyclic Diarylheptanoids from Platycarya strobilacea
Source: Molecules. 2020 Dec 20;25(24):6034. doi: 10.3390/molecules25246034 (PMC7766178; doi:10.3390/molecules25246034)
Supplement: Supplementary file 1 [file molecules-25-06034-s001.pdf]

SUPPLEMENTARY MATERIAL

New Cyclic Diarylheptanoids from *Platycarya strobilacea*

Wen-bing Ding [1, 2], Rui-yuan Zhao[3], Guan-hua Li [2], Bing-lei Liu [3], Hua-liang He [2], Lin Qiu [2], Jin Xue[2] and You-zhi Li [1, 2*]

[1] Hunan Provincial Engineering & Technology Research Center for Biopesticide and Formulation Processing, Hunan Agricultural University, Changsha 410128, China; dingwenb119@ hunau.edu.cn (W. Ding);

[2] National Research Center of Engineering & Technology for Utilization of Botanical Functional Ingredients, Hunan Agricultural University, Changsha 410128, China; 317690477@qq.com (G. Li); hhl_1234@126.com (H. He); qiulin@hunau.edu.cn (L. Qiu); xuejin@hunau.edu.cn (J. Xue)

[3] Hunnan Cotton Science Institute, Changde 415100, China; zhaoruiyuan@vip.sina.com (R. Zhao); lbl5155@163.com (B. Liu)

* Correspondence: liyouzhi@ hunau.edu.cn; Tel.: +8613808477468 (Y. Li)

Figure list: (HR-ESIMS, 1D and 2D NMR spectra of compound **1**–**5**)

Figure S1. HR-ESIMS of compound **1**	Figure S21 HR-ESIMS of compound **4**
Figure S2. ^{1}H-NMR of compound **1**	Figure S22. ^{1}H-NMR of compound **4**
Figure S3. ^{13}C-NMR of compound **1**	Figure S22. ^{13}C-NMR of compound **4**
Figure S4. ^{1}H-^{1}H COSY of compound **1**	Figure S24. ^{1}H-^{1}H COSY of compound **4**
Figure S5. HSQC of compound **1**	Figure S24. HSQC of compound **4**
Figure S6. HMBC of compound **1**	Figure S26. HMBC of compound **4**
Figure S7. NOESY of compound **1**	Figure S27. HR-ESIMS of compound **5**
Figure S8. HR-ESIMS of compound **2**	Figure S28. ^{1}H-NMR of compound **5**
Figure S9. ^{1}H-NMR of compound **2**	Figure S29. ^{13}C-NMR of compound **5**
Figure S10. ^{13}C-NMR of compound **2**	Figure S30. ^{1}H-^{1}H COSY of compound **5**
Figure S11. ^{1}H-^{1}H COSY of compound **2**	Figure S31. HSQC of compound **5**
Figure S12. HSQC of compound **2**	Figure S32. HMBC of compound **5**
Figure S13. HMBC of compound **2**	Figure S33. NOESY of compound **5**
Figure S14. NOESY of compound **2**	
Figure S15. HR-ESIMS of compound **3**	
Figure S16. ^{1}H-NMR of compound **3**	
Figure S17. ^{13}C-NMR of compound **3**	
Figure S18. ^{1}H-^{1}H COSY of compound **3**	
Figure S19. HSQC of compound **3**	
Figure S20. HMBC of compound **3**	

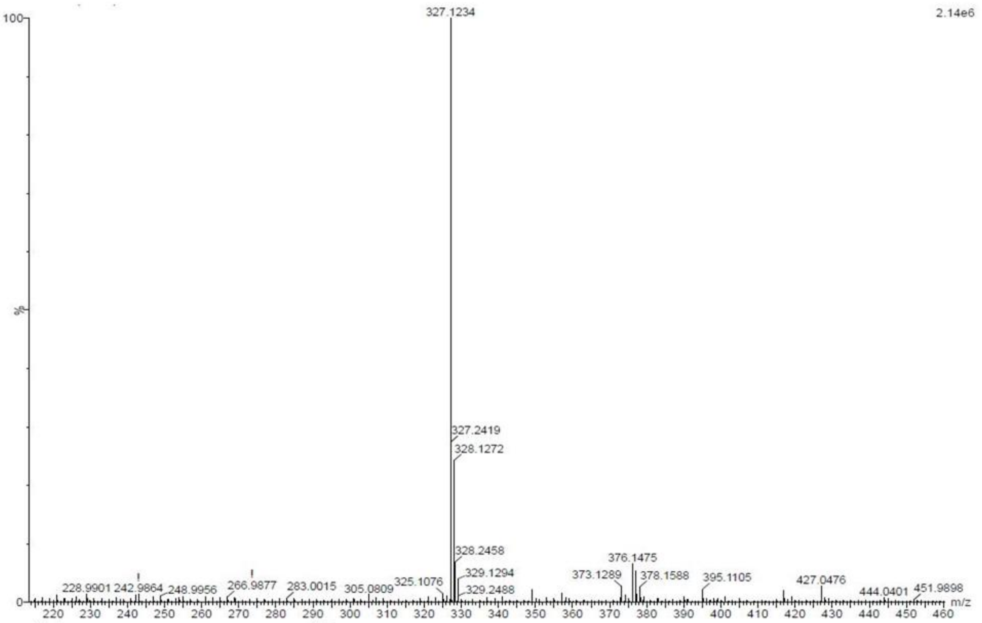

Minimum:				-1.5				
Maximum:		20.0	5.0	50.0				
Mass	Calc. Mass	mDa	PPM	DBE	i-FIT	Norm	Conf(%)	Formula
327.1234	327.1232	0.2	0.6	10.5	1160.2	n/a	n/a	C19 H19 O5

Figure S1. HR-ESIMS of compound **1**

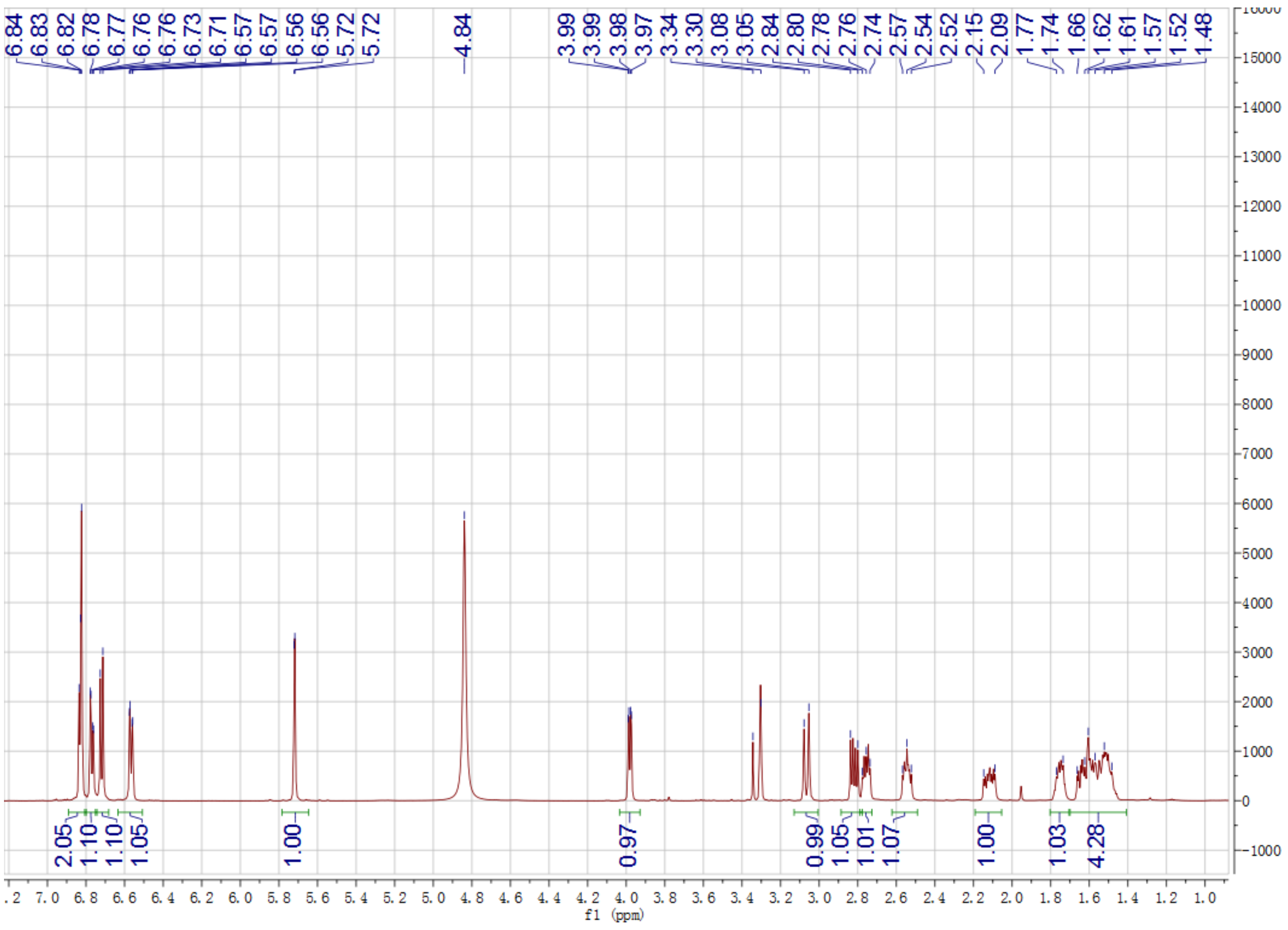

Figure S2. ^{1}H-NMR of compound **1**

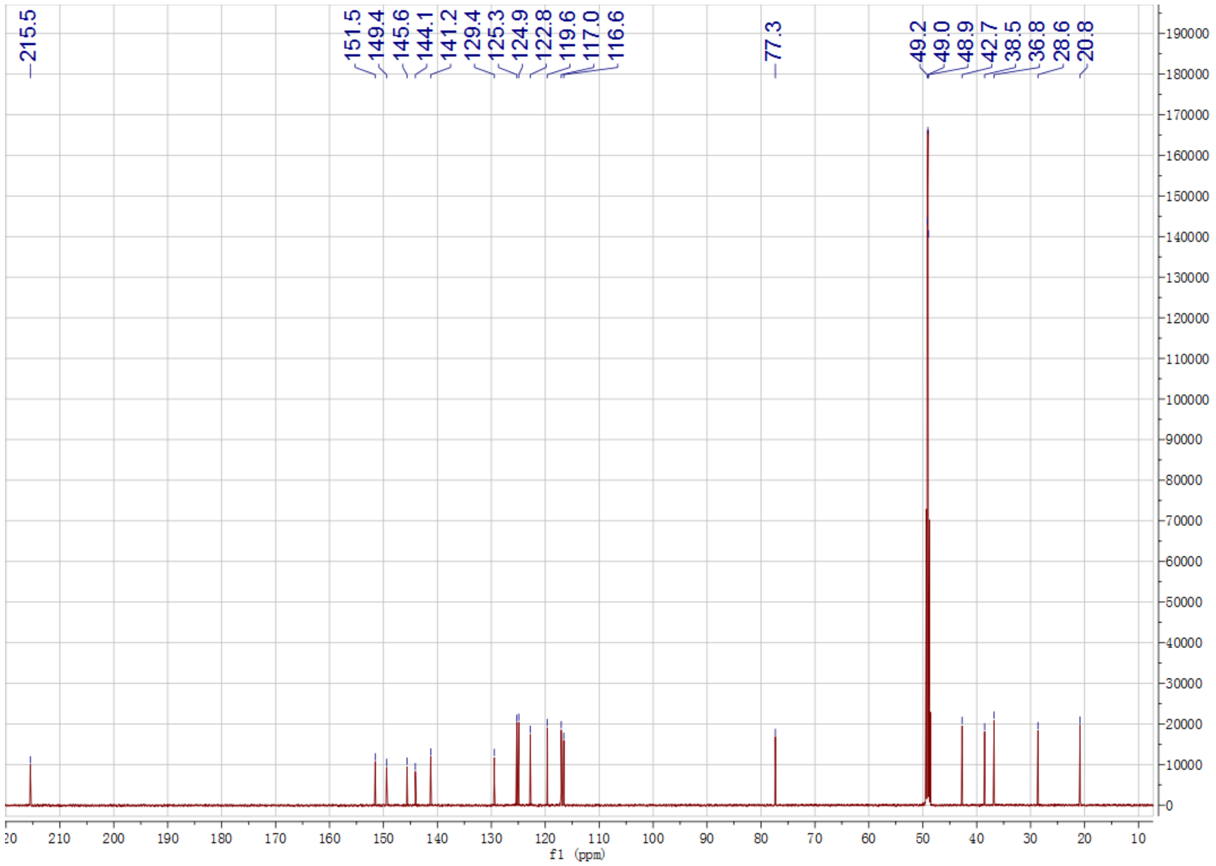

Figure S3. ^{13}C-NMR of compound **1**

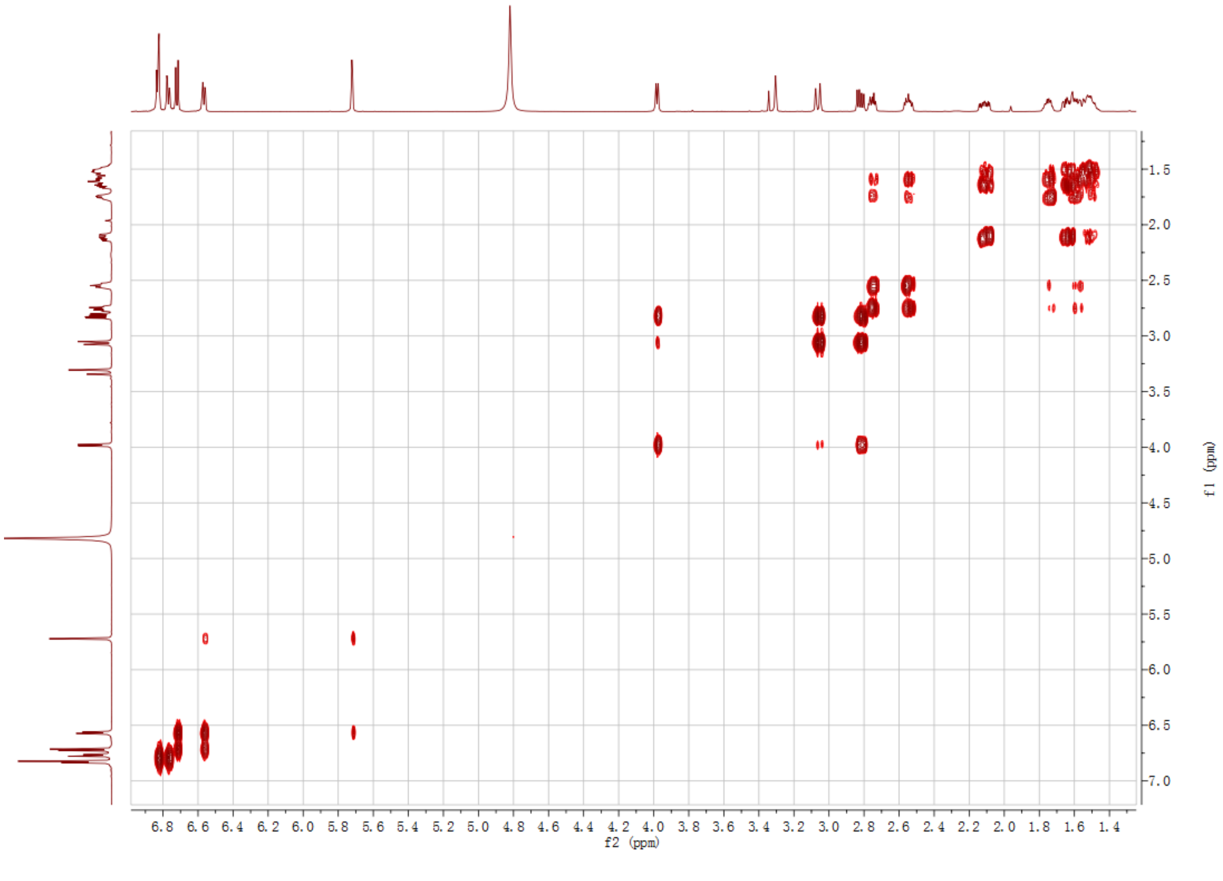

Figure S4. ^{1}H-^{1}H COSY of compound **1**

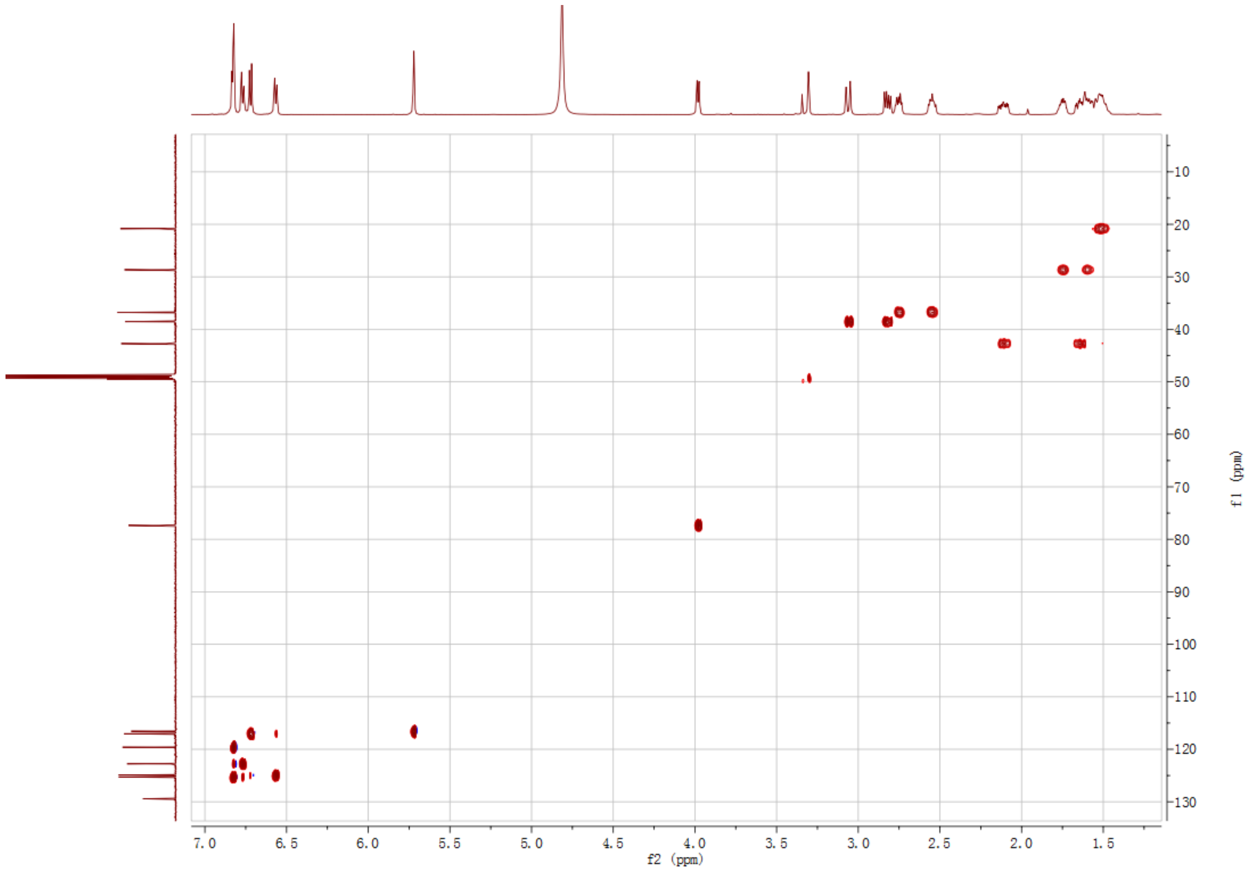

Figure S5. HSQC of compound **1**

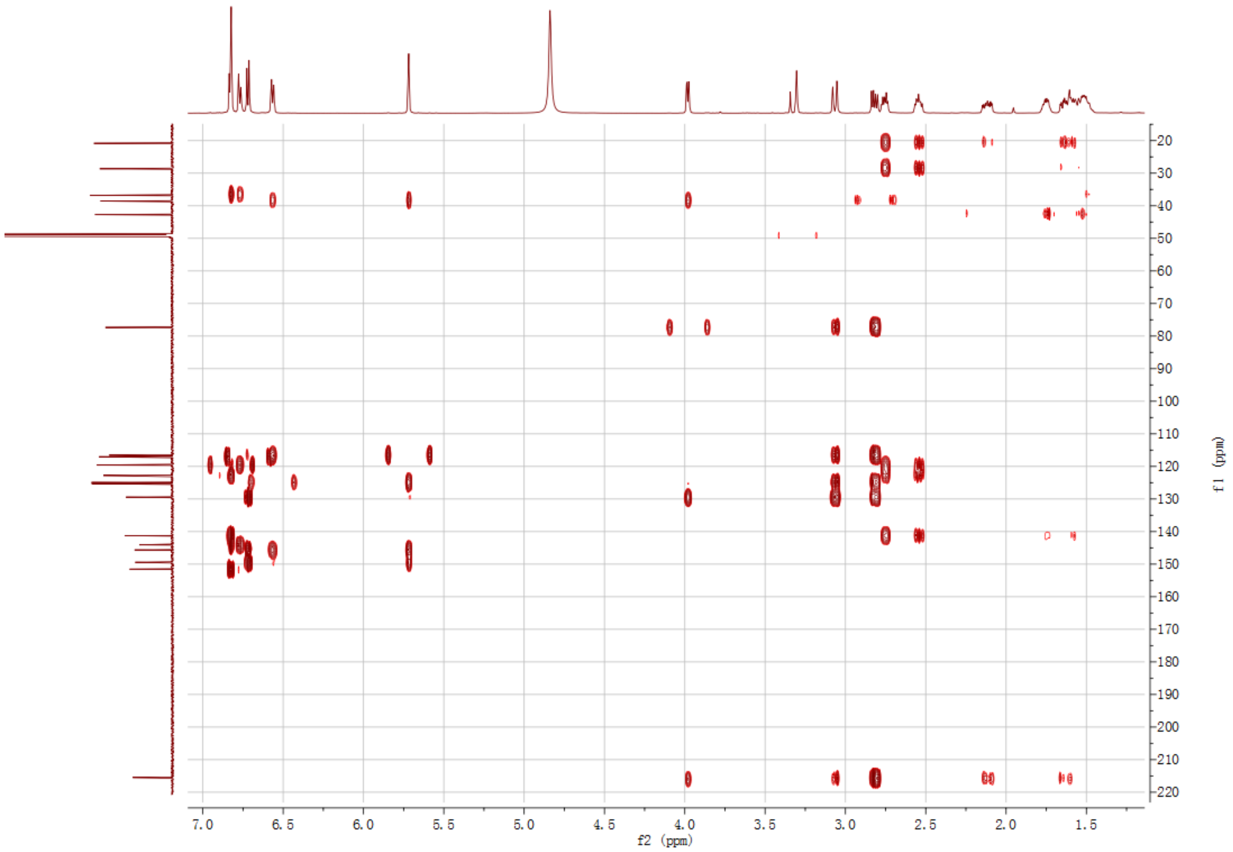

Figure S6. HMBC of compound **1**

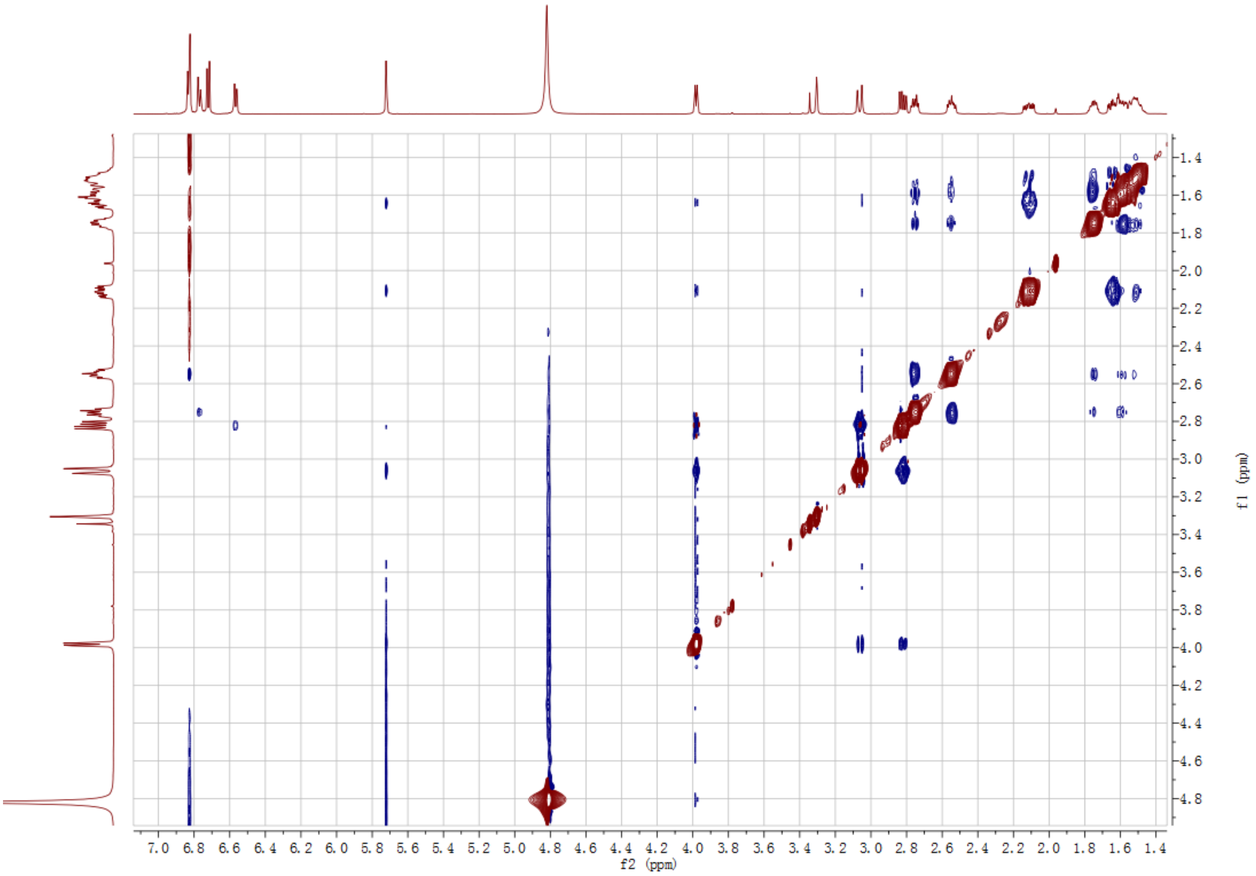

Figure S7. NOESY of compound **1**

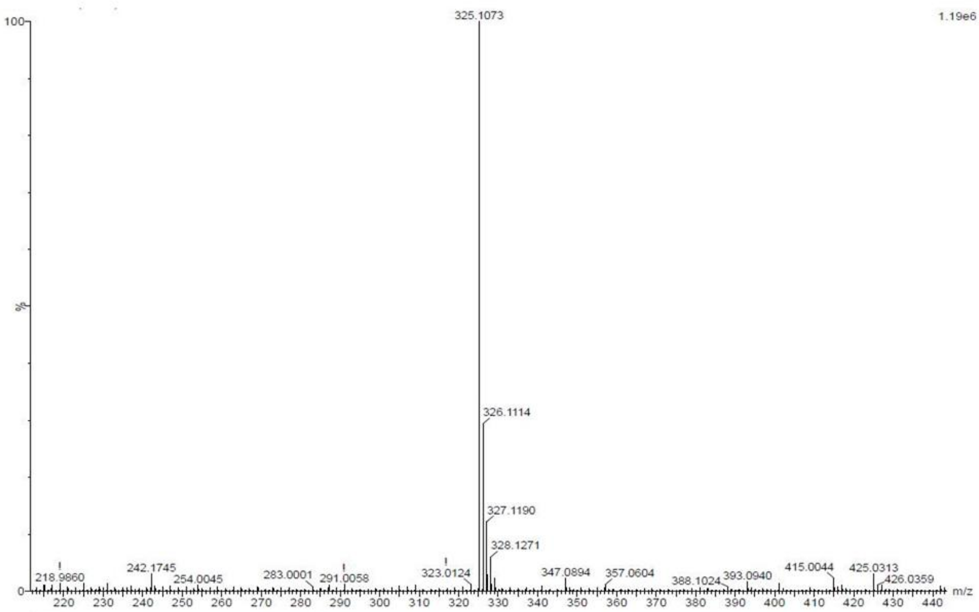

Minimum:				-1.5				
Maximum:		20.0	5.0	50.0				
Mass	Calc. Mass	mDa	PPM	DBE	i-FIT	Norm	Conf(%)	Formula
325.1073	325.1076	-0.3	-0.9	11.5	1161.8	n/a	n/a	C19 H17 O5

Figure S8. HR-ESIMS of compound **2**

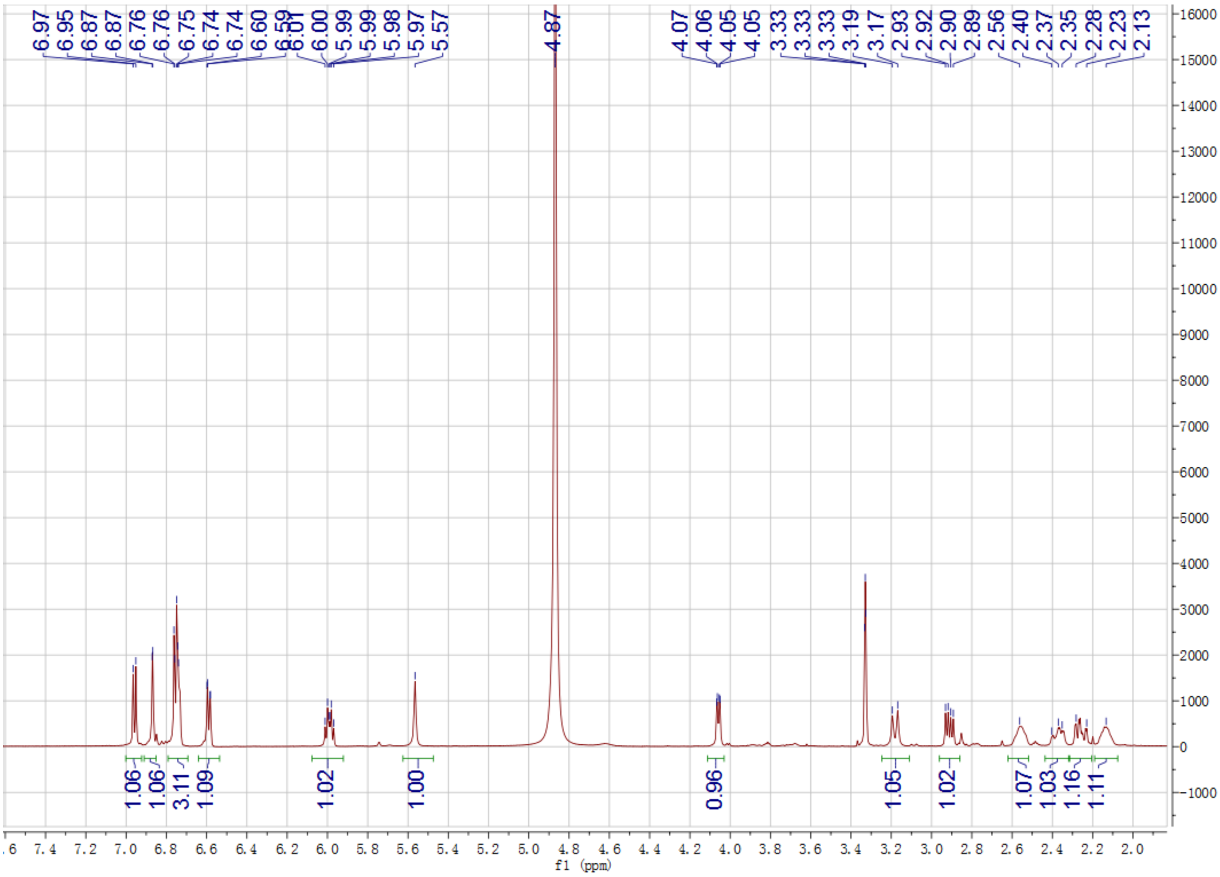

Figure S9. ^{1}H-NMR of compound **2**

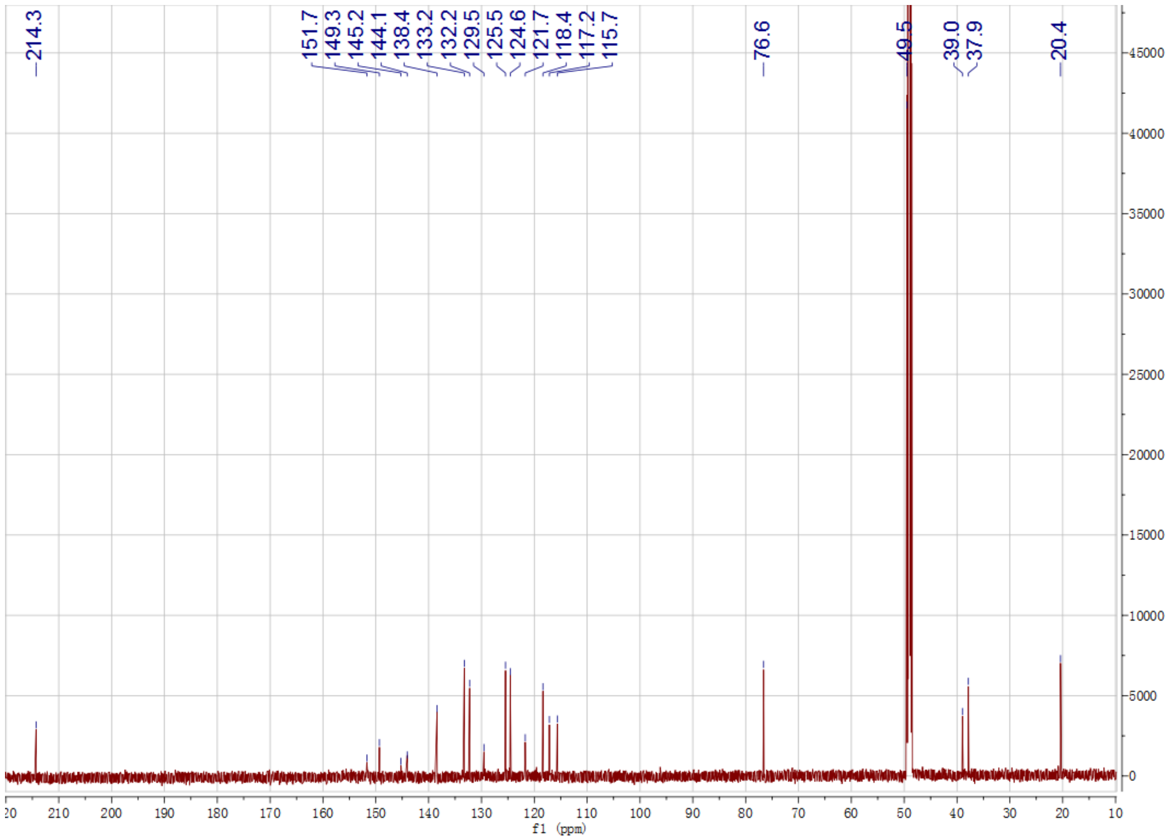

Figure S10. ^{13}C-NMR of compound **2**

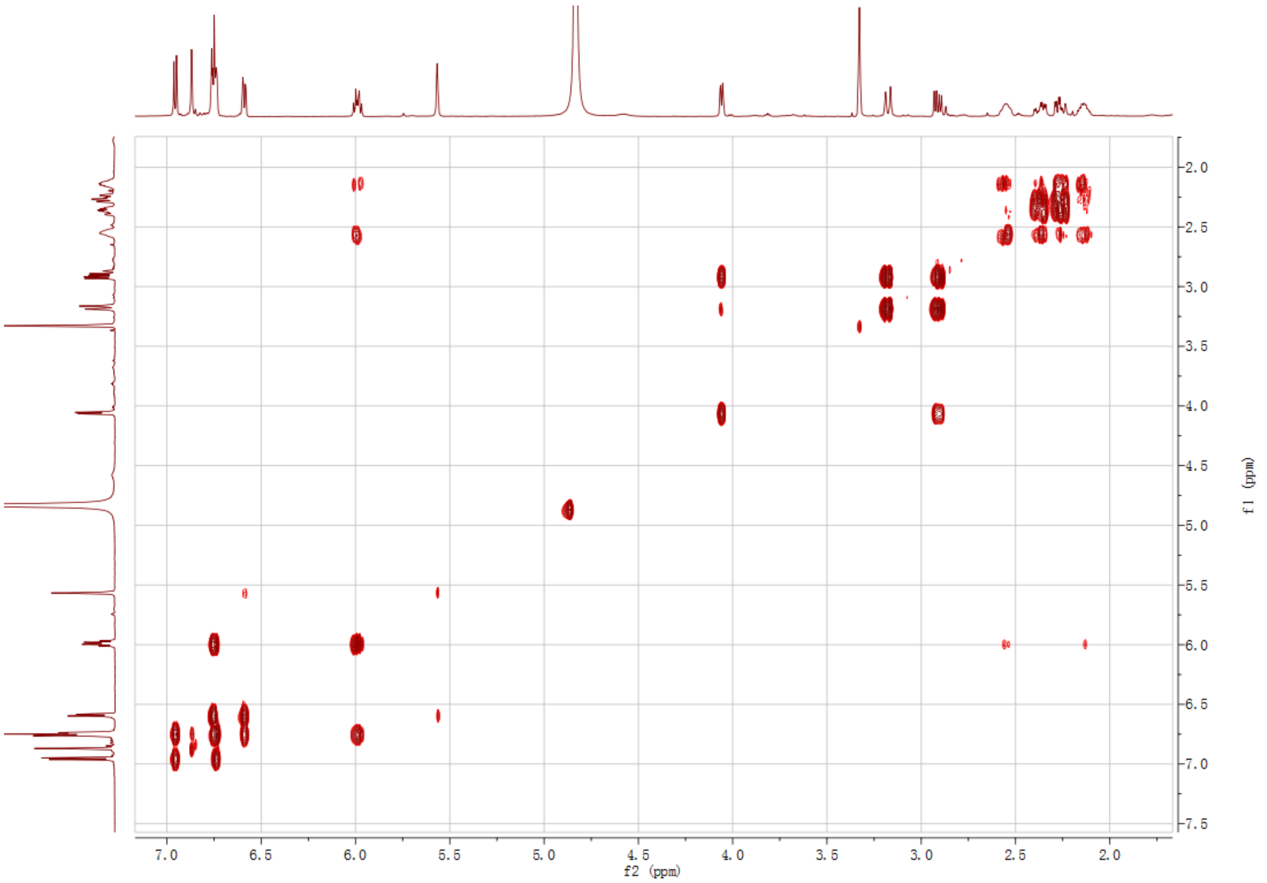

Figure S11. ^{1}H-^{1}H COSY of compound **2**

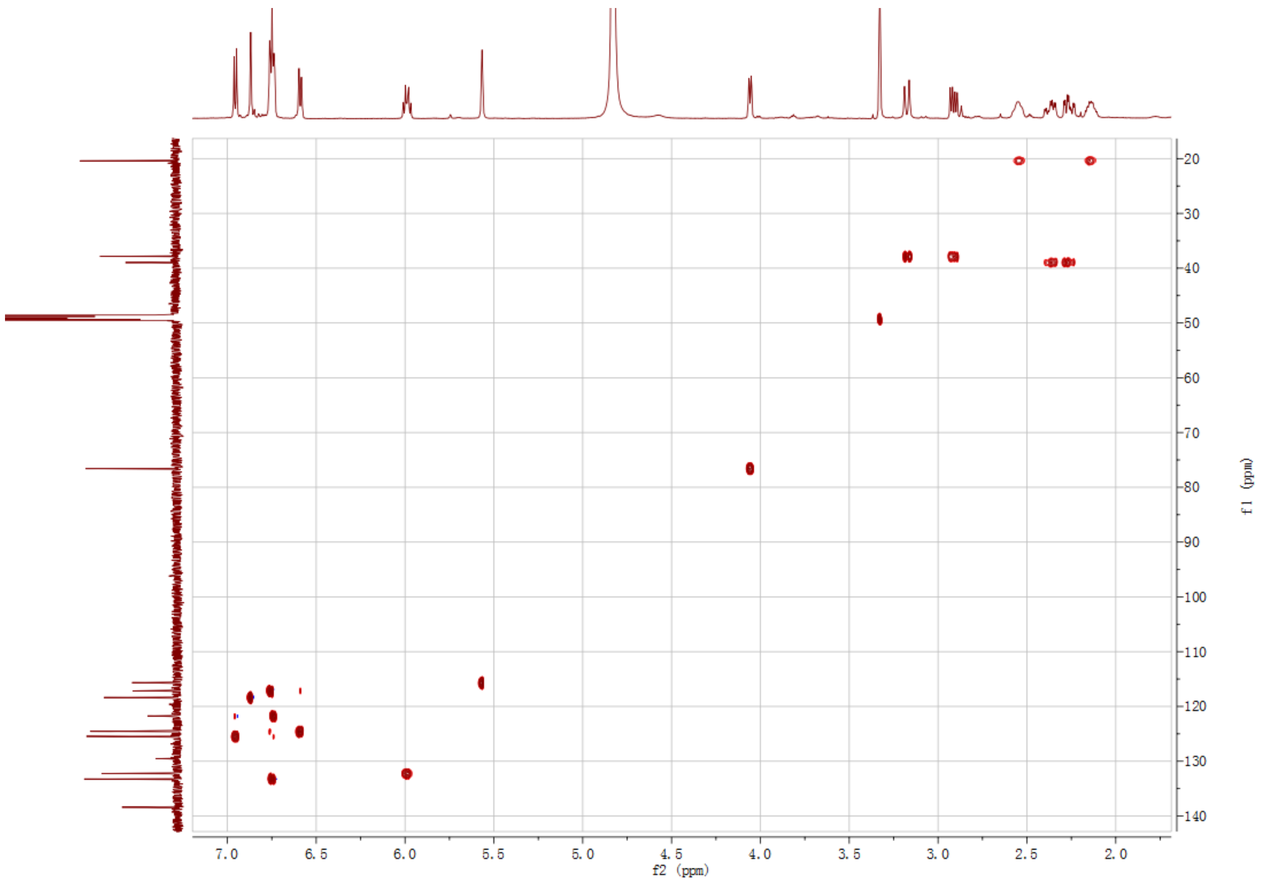

Figure S12. HSQC of compound **2**

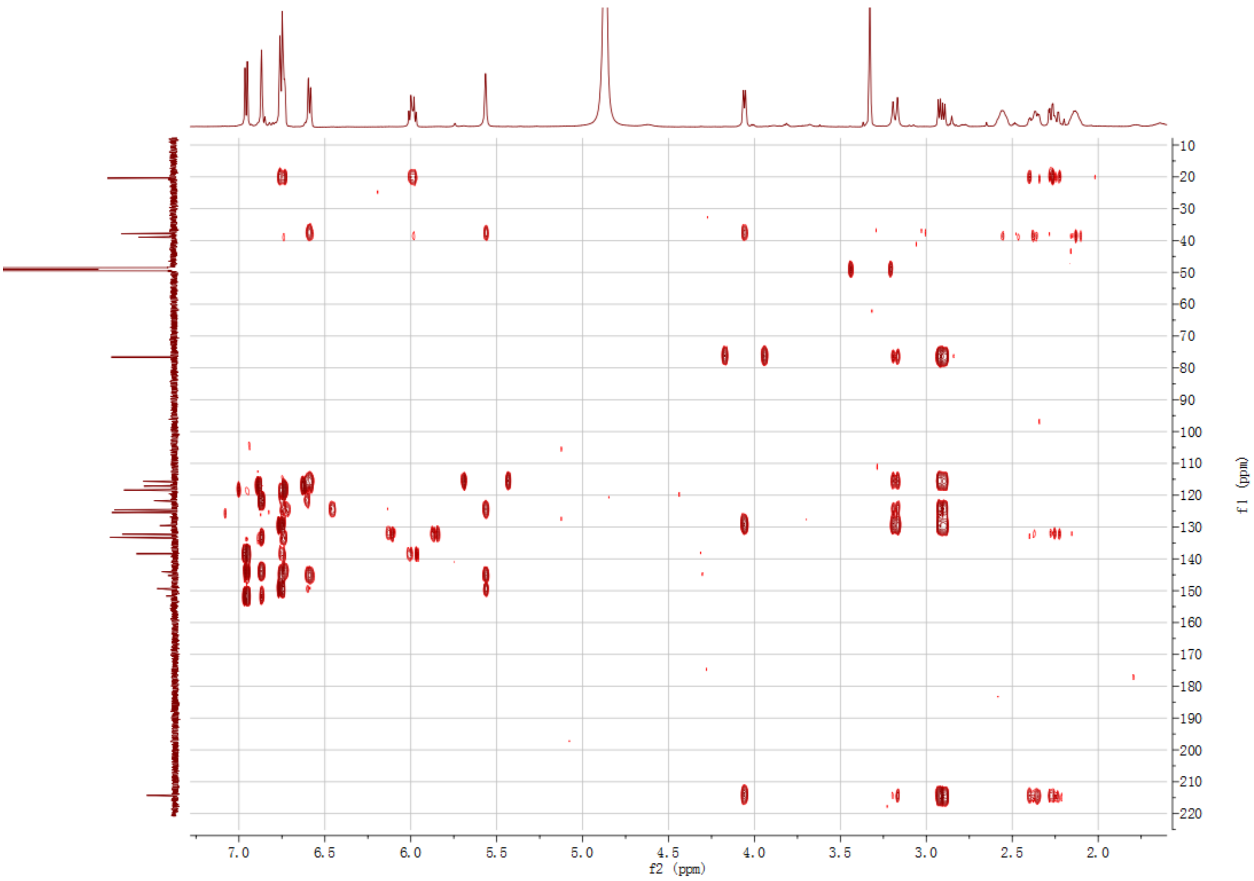

Figure S13. HMBC of compound **2**

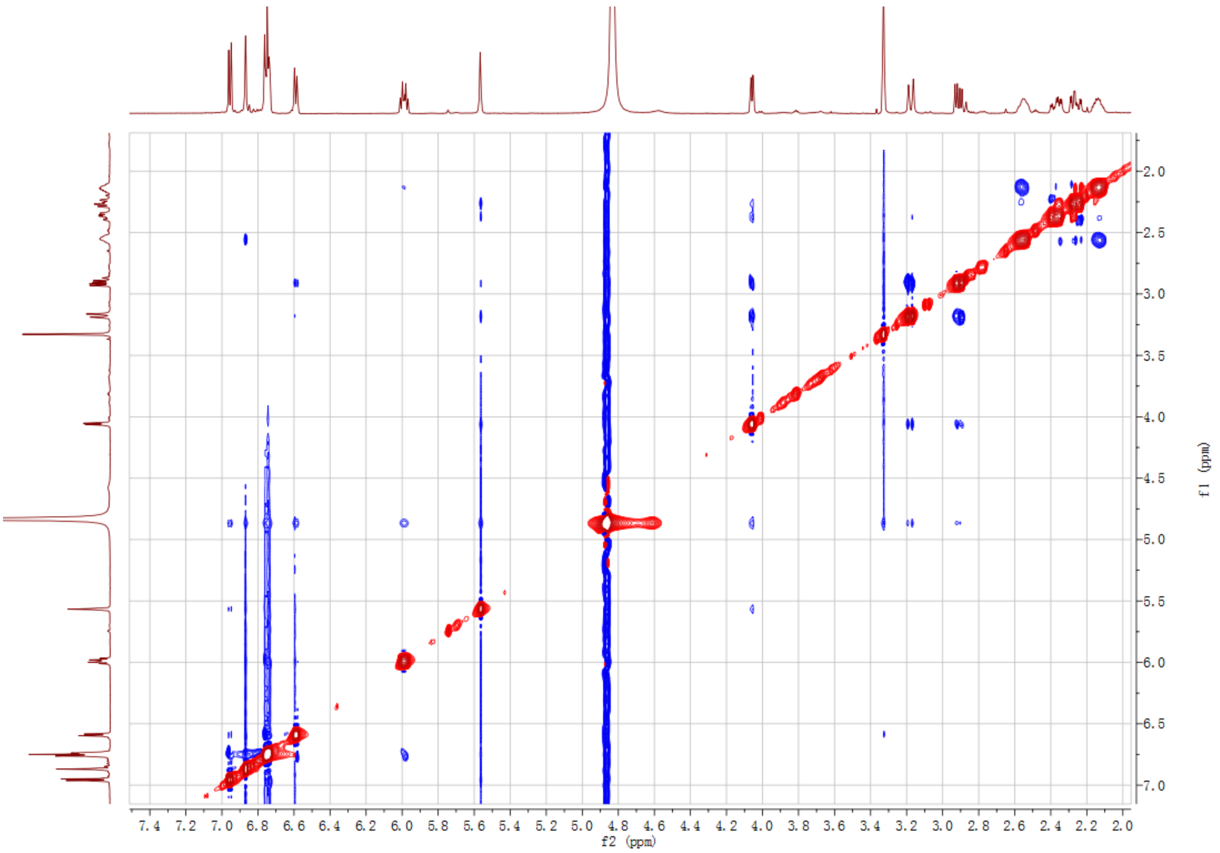

Figure S14. NOESY of compound **2**

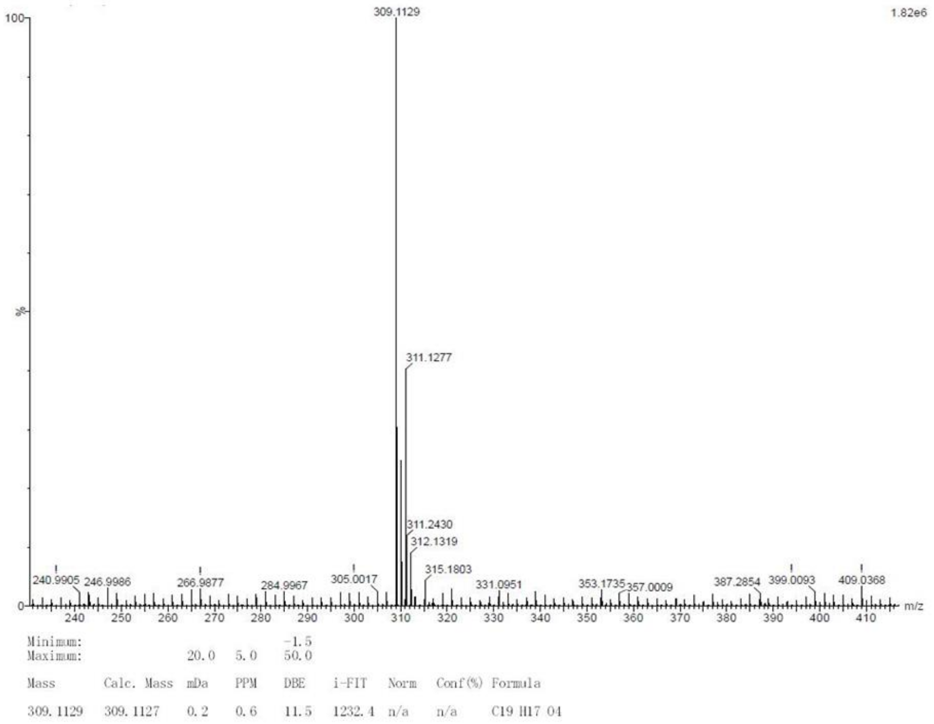

Figure S15. HR-ESIMS of compound **3**

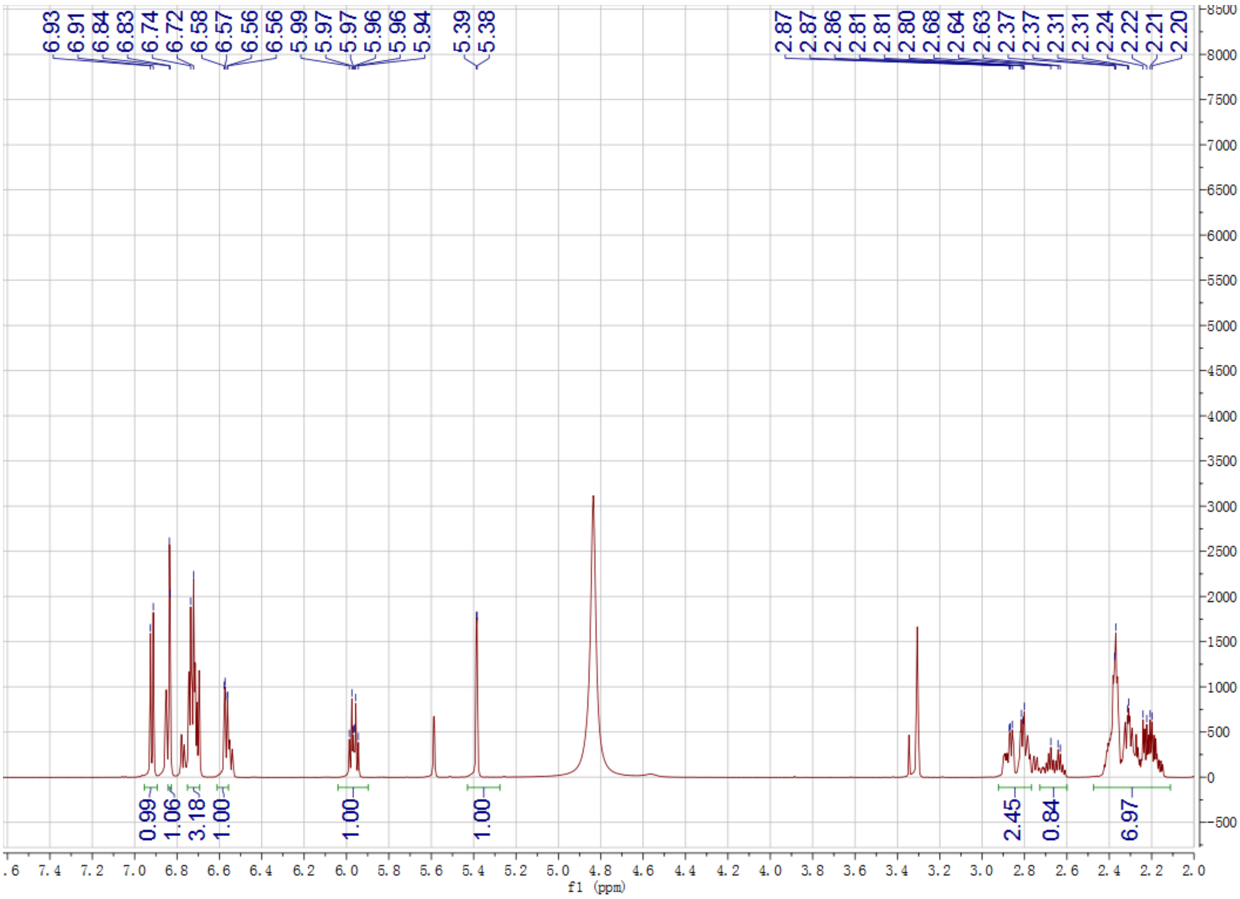

Figure S16. ^{1}H-NMR of compound **3**

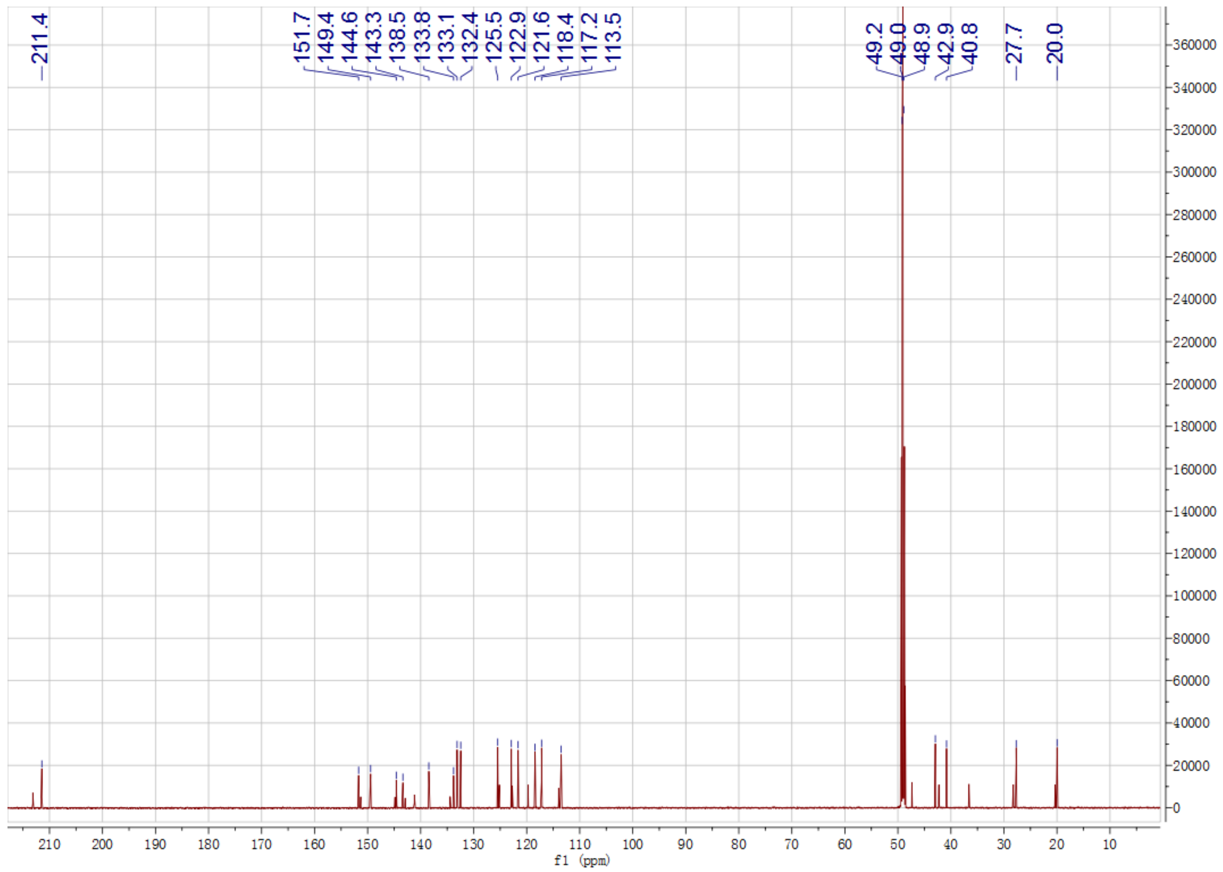

Figure S17. ^{13}C-NMR of compound **3**

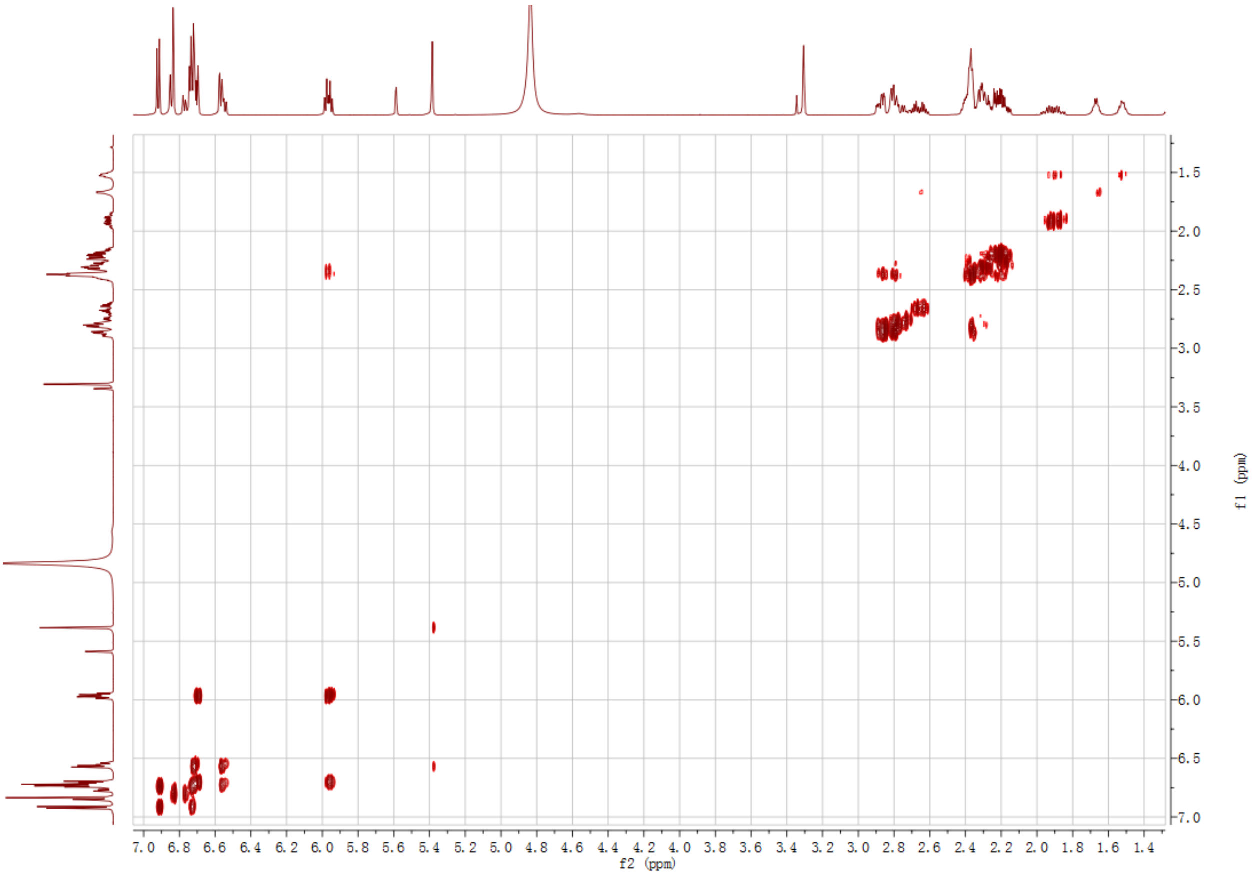

Figure S18. ^{1}H-^{1}H COSY of compound **3**

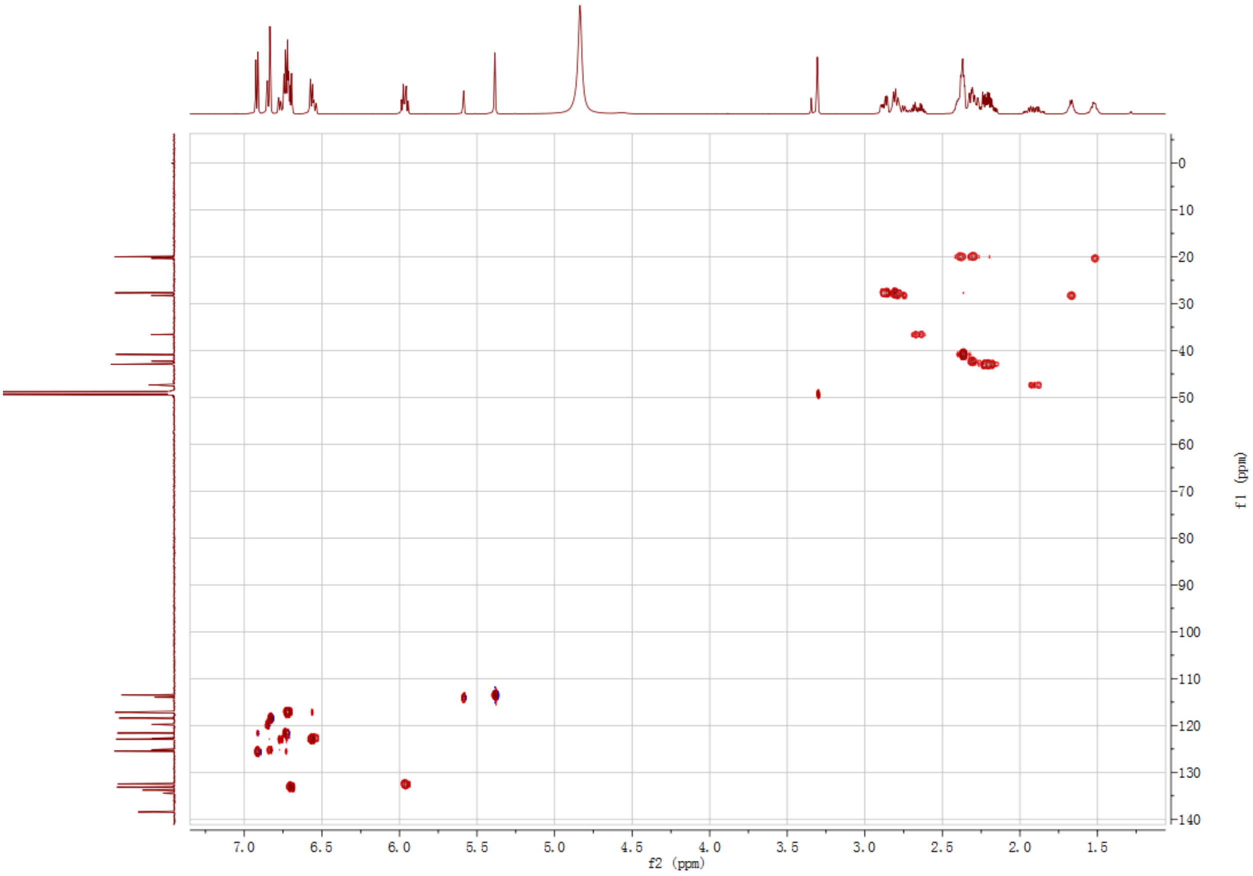

Figure S19. HSQC of compound **3**

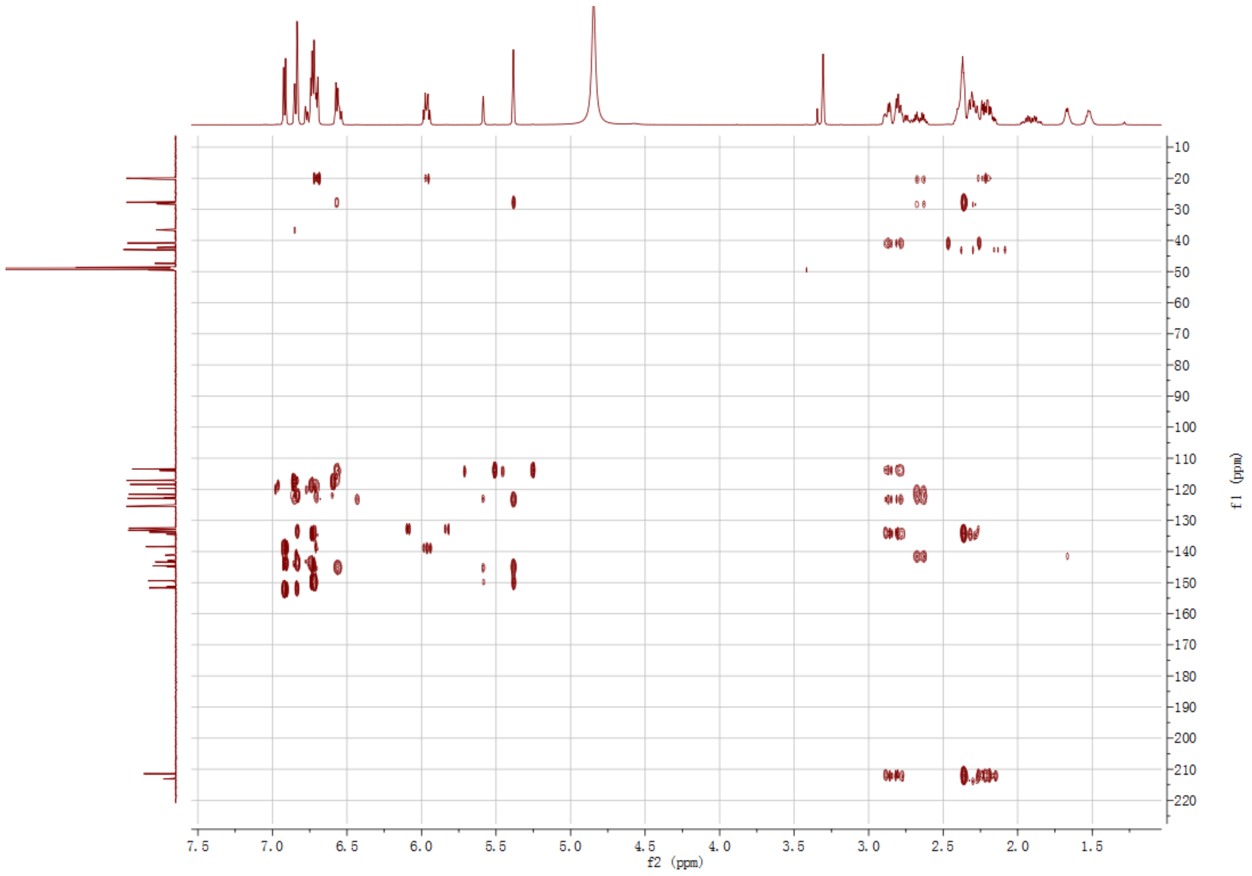

Figure S20. HMBC of compound **3**

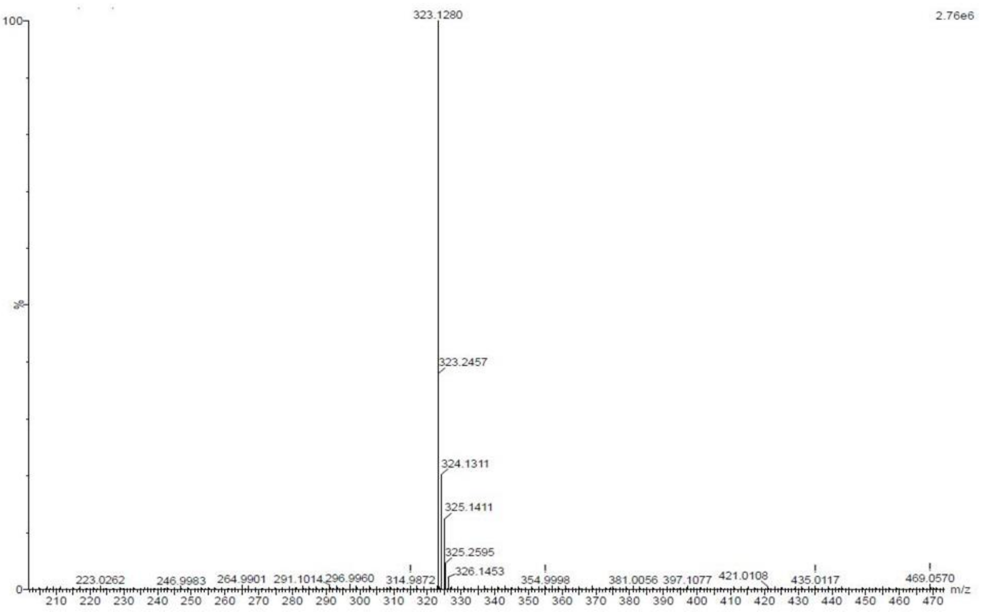

	Calc. Mass	mDa	PPM	DBE	i-FIT	Norm	Conf(%)	Formula
Minimum:				-1.5				
Maximum:		20.0	5.0	50.0				
Mass	Calc. Mass	mDa	PPM	DBE	i-FIT	Norm	Conf(%)	Formula
323.1280	323.1283	-0.3	-0.9	11.5	1301.4	n/a	n/a	C20 H19 O4

Figure S21 HR-ESIMS of compound **4**

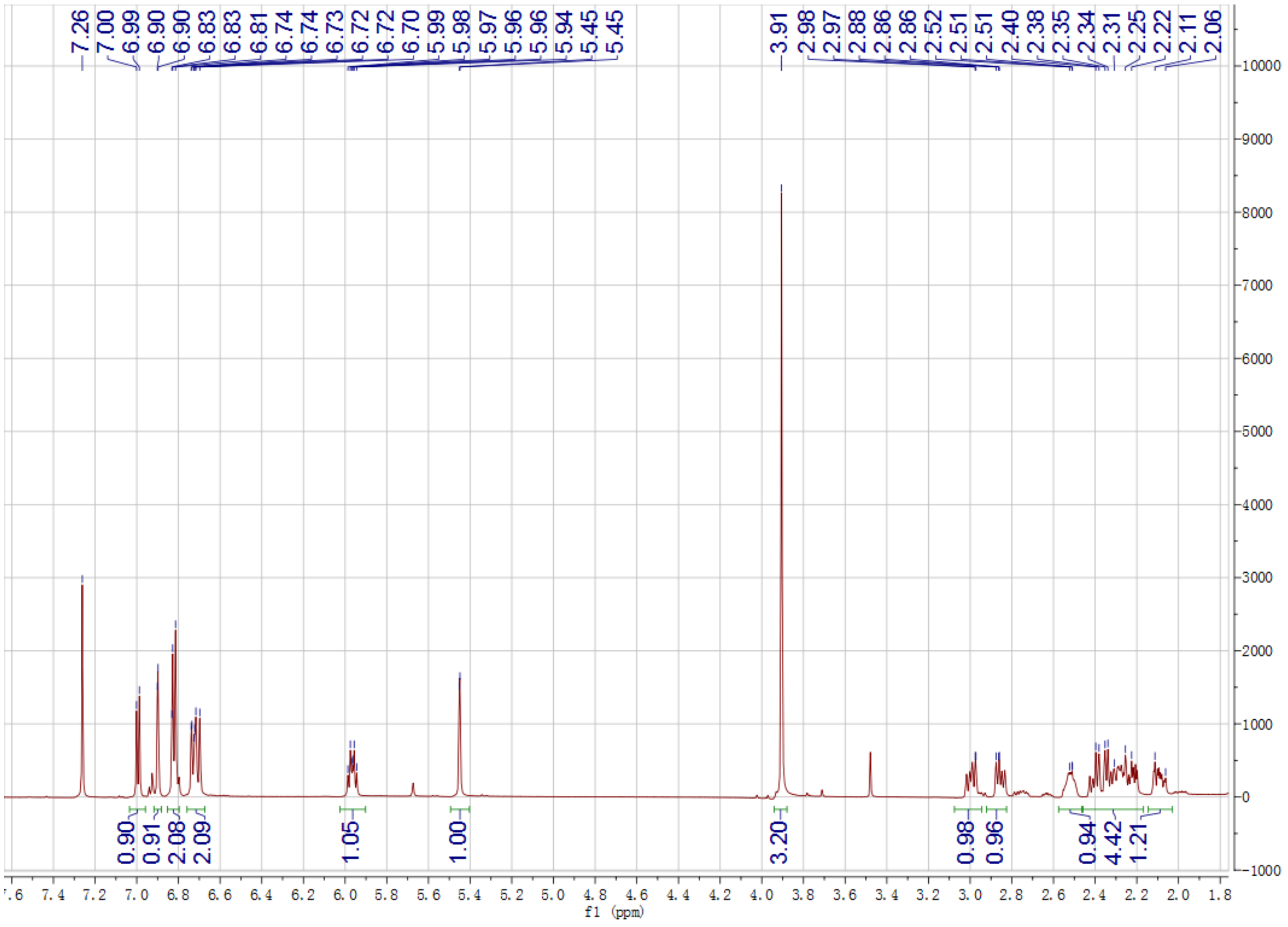

Figure S22. ^{1}H-NMR of compound **4**

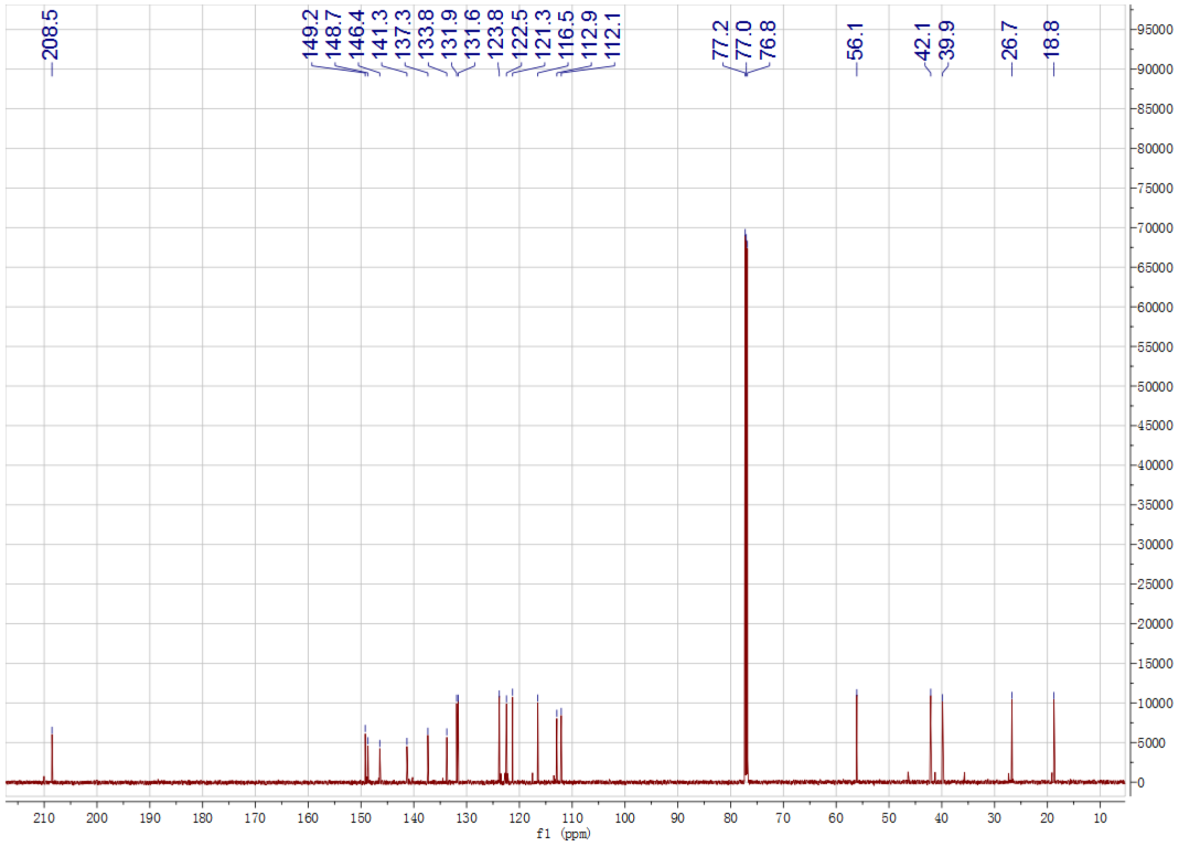

Figure S22. ^{13}C-NMR of compound **4**

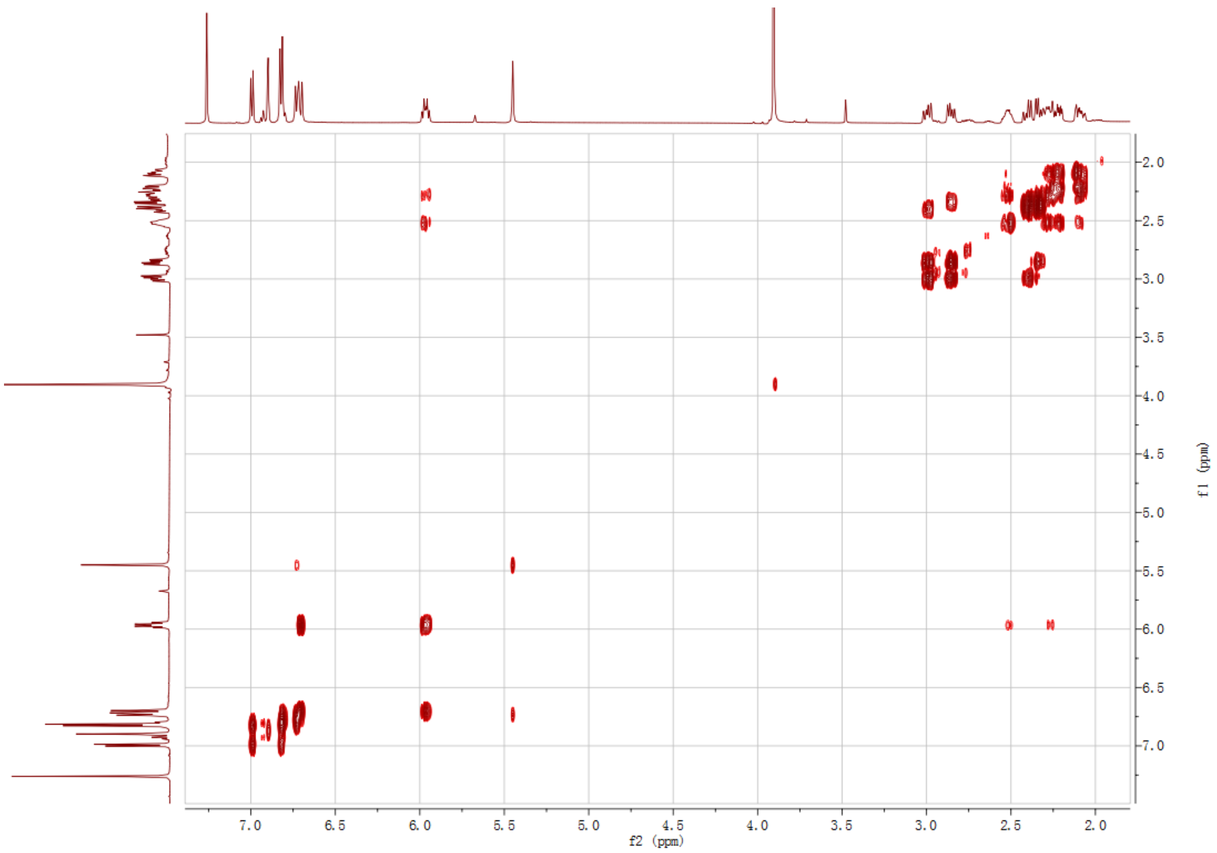

Figure S24. ^{1}H-^{1}H COSY of compound **4**

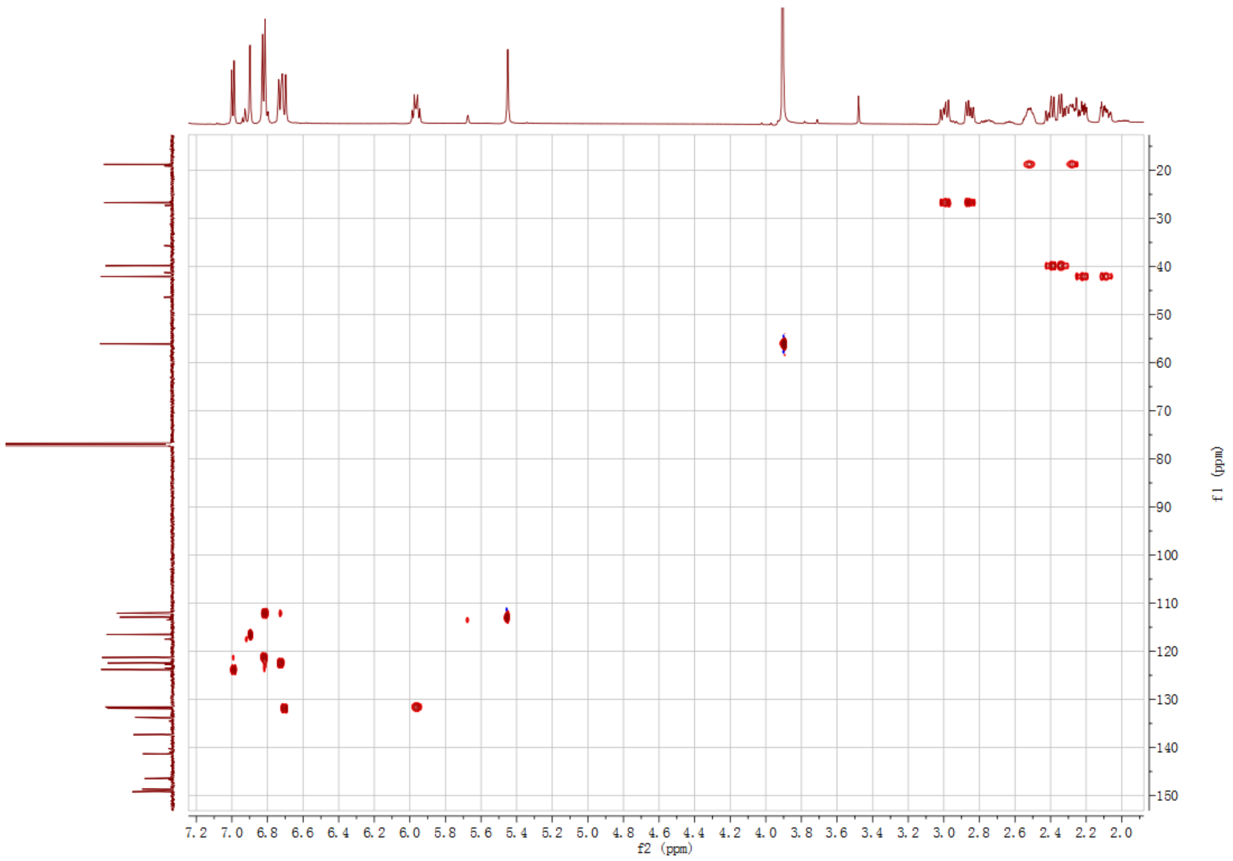

Figure S24. HSQC of compound **4**

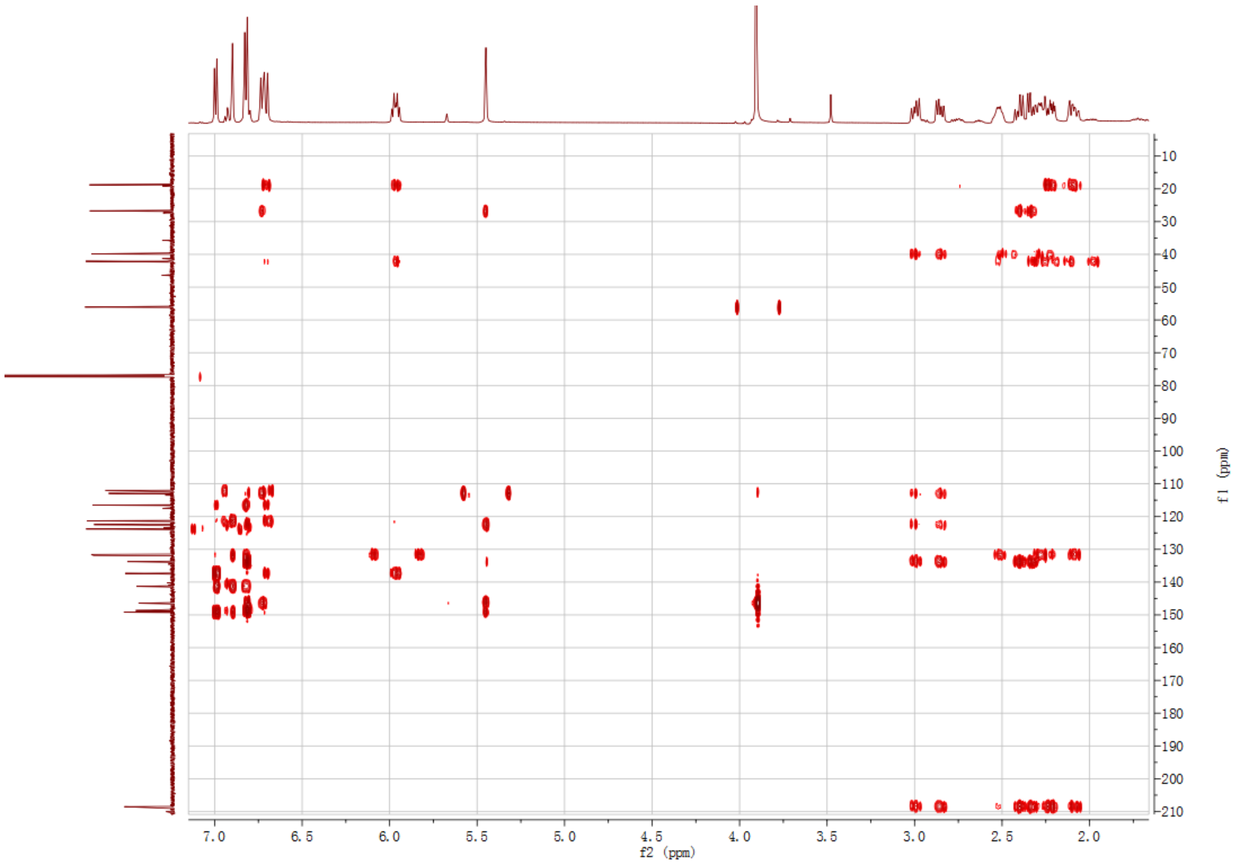

Figure S26. HMBC of compound **4**

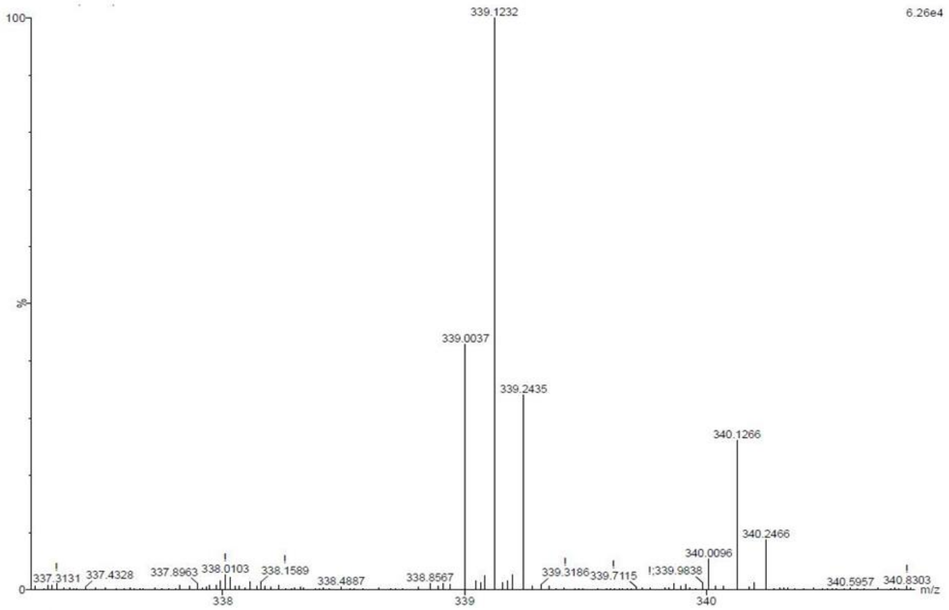

	Calc. Mass	mDa	PPM	DBE	i-FIT	Norm	Conf(%)	Formula
Minimum:				-1.5				
Maximum:		20.0	5.0	50.0				
Mass	Calc. Mass	mDa	PPM	DBE	i-FIT	Norm	Conf(%)	Formula
339.1232	339.1232	0.0	0.0	11.5	692.0	n/a	n/a	C20 H19 O5

Figure S27. HR-ESIMS of compound **5**

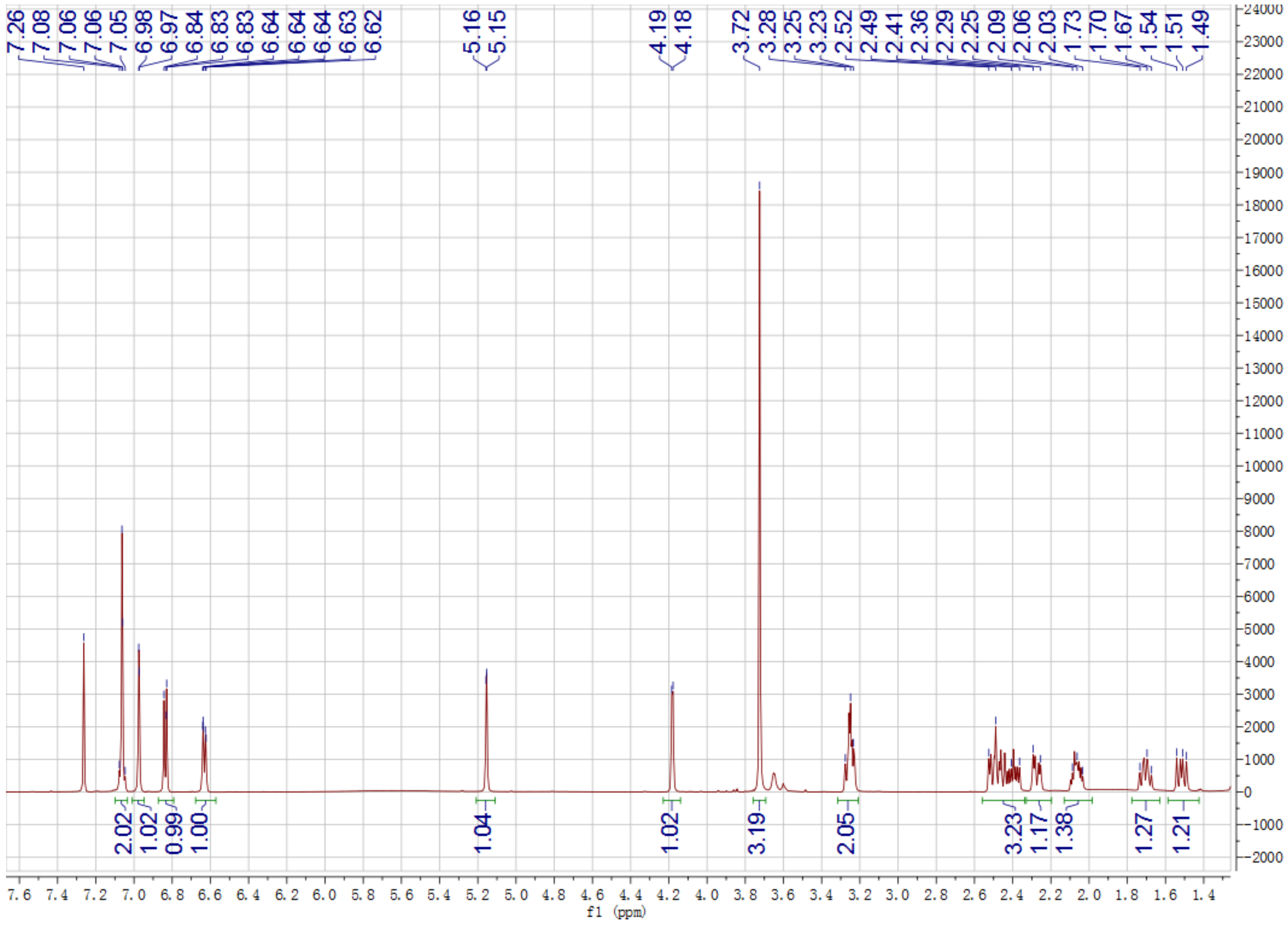

Figure S28. ^{1}H-NMR of compound **5**

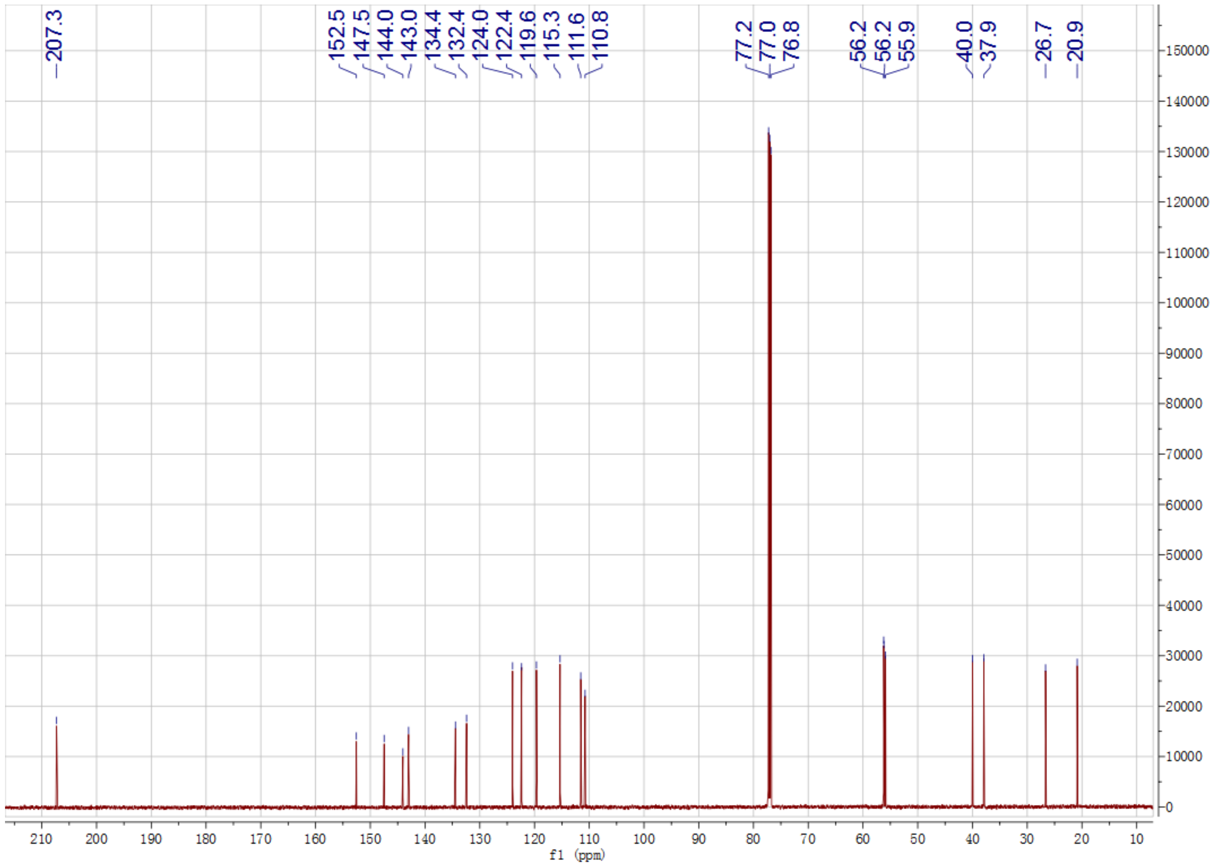

Figure S29. ^{13}C-NMR of compound **5**

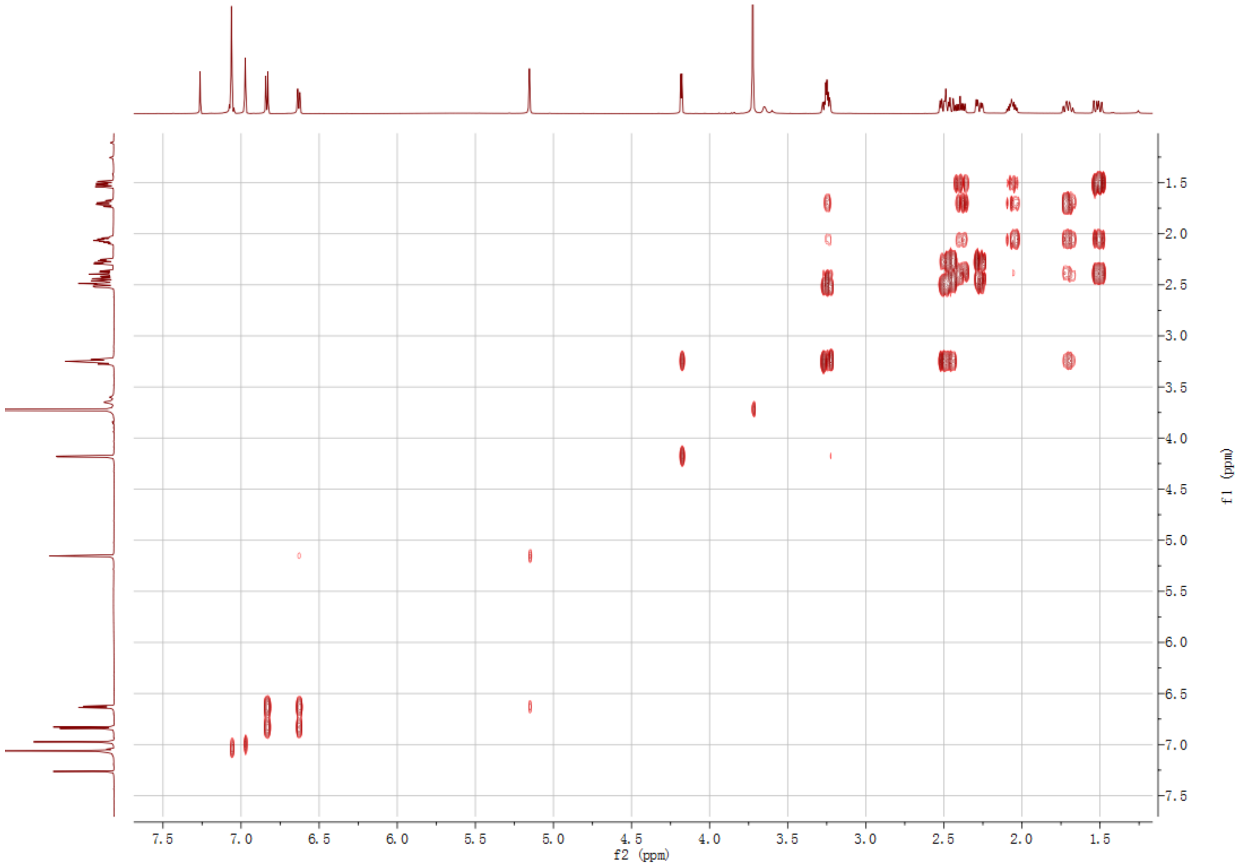

Figure S30. ^{1}H-^{1}H COSY of compound **5**

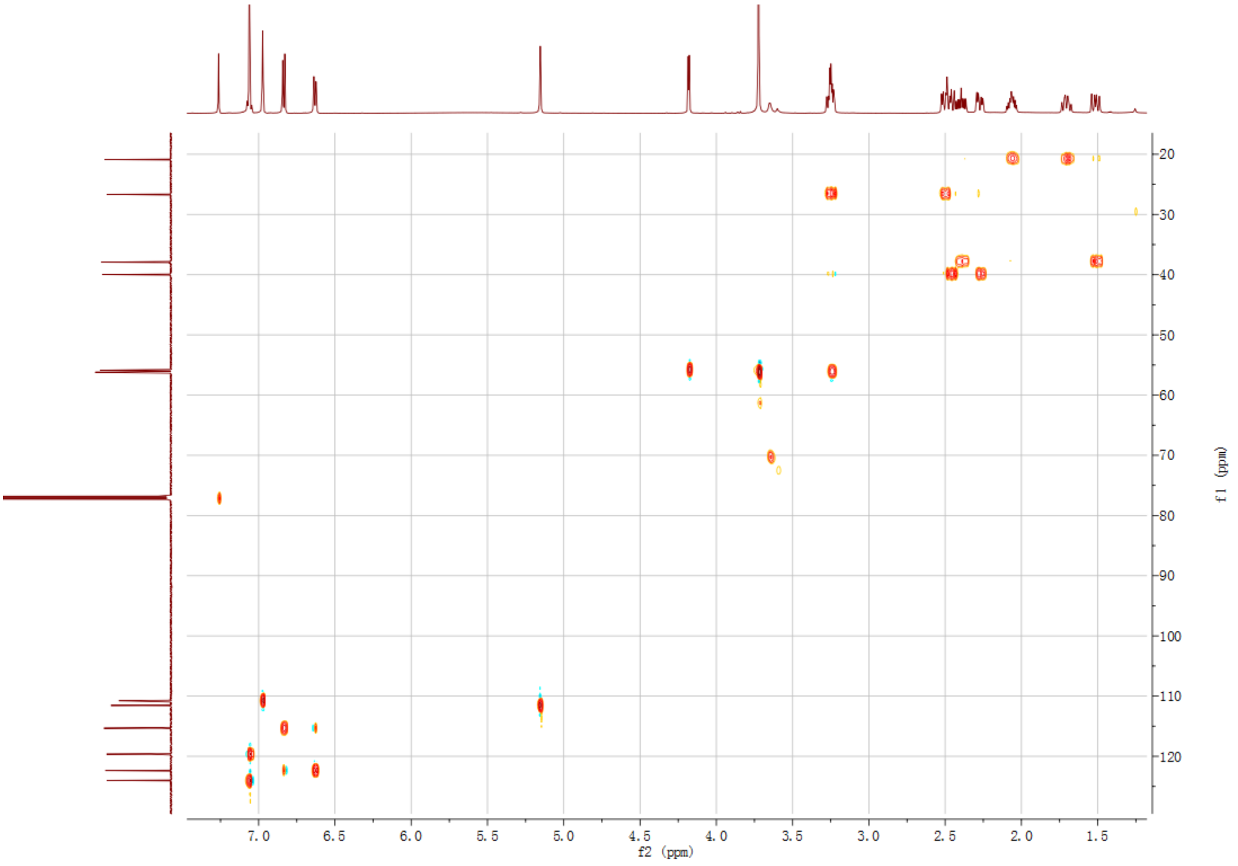

Figure S31. HSQC of compound **5**

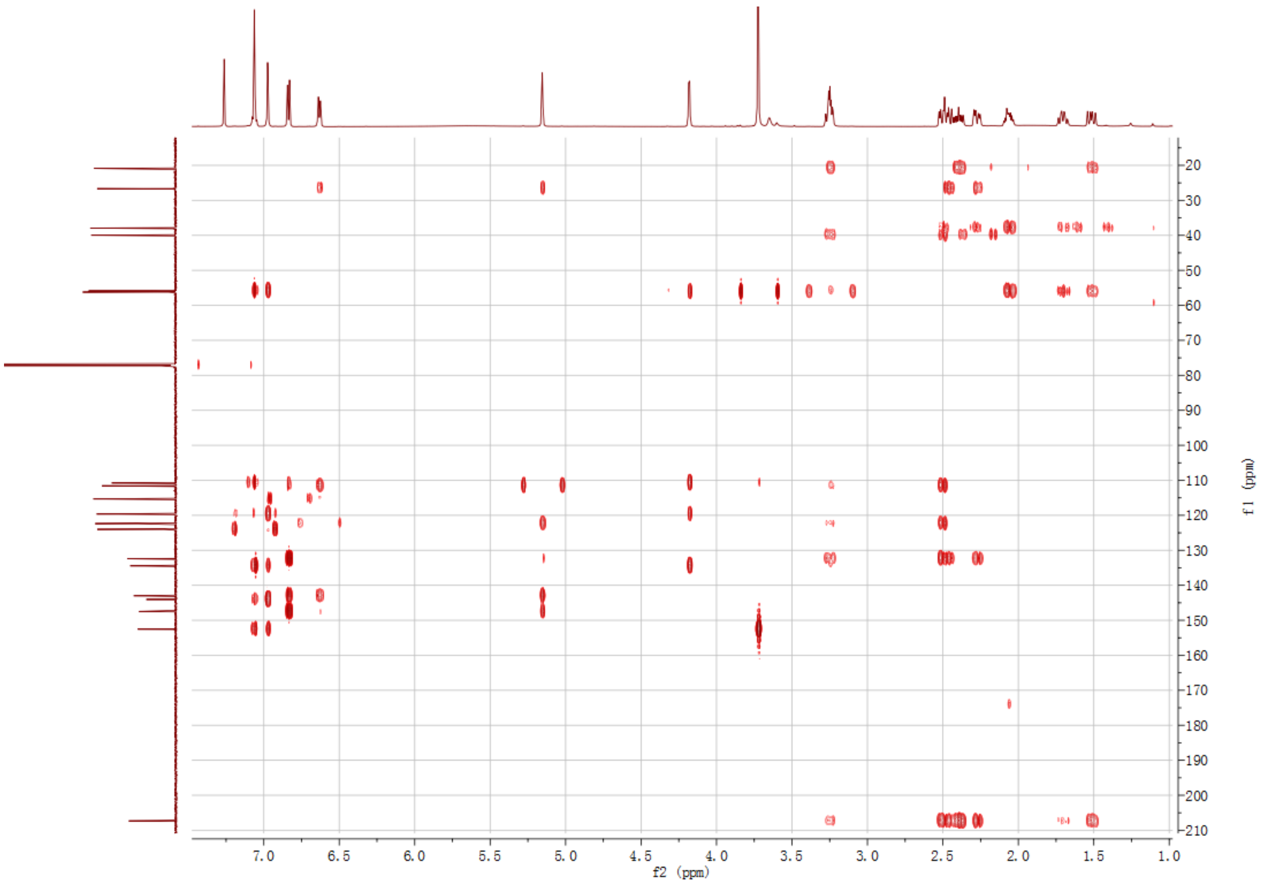

Figure S32. HMBC of compound **5**

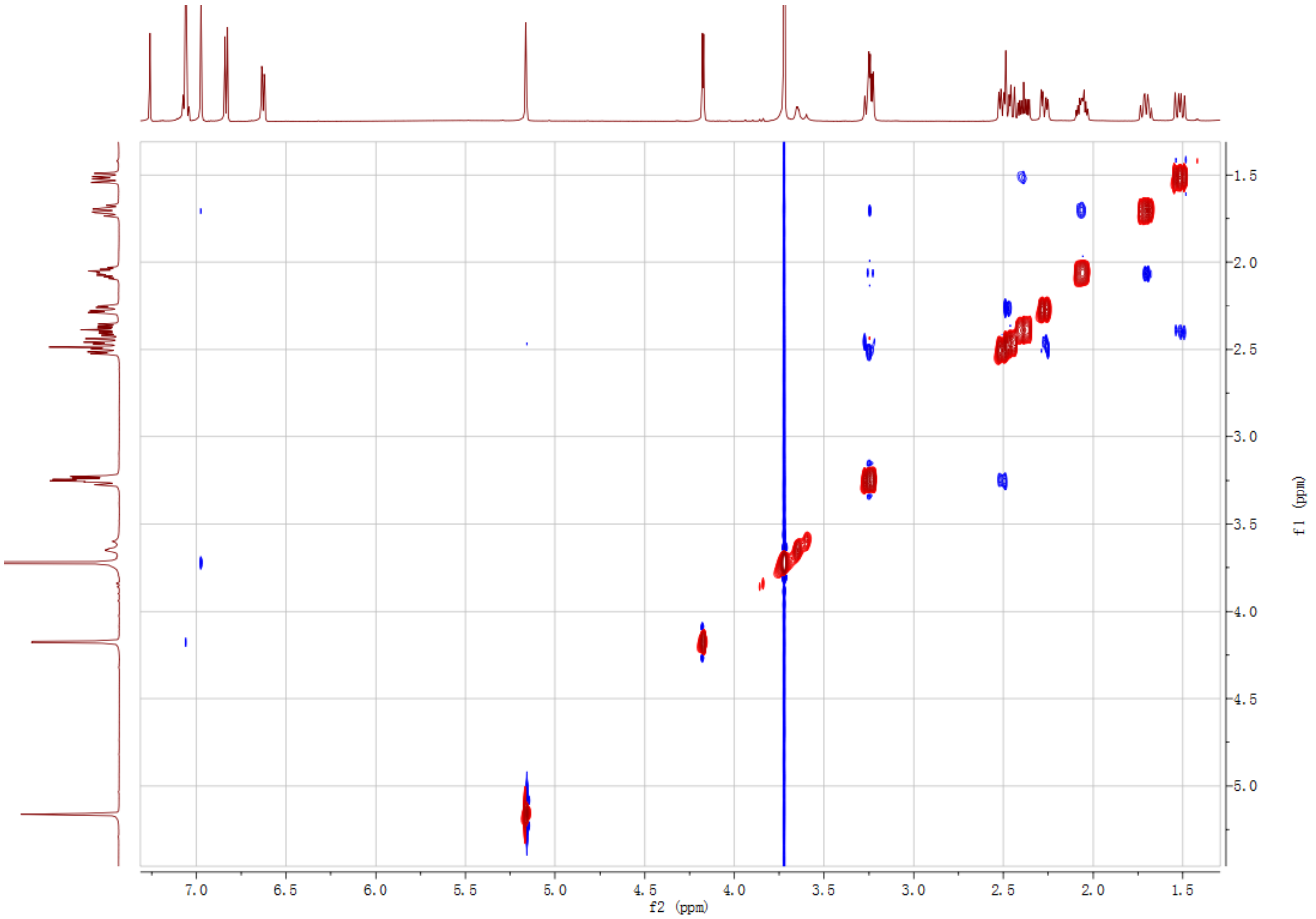

Figure S33. NOESY of compound **5**
